# Optimising professional support for doctors who experience work performance issues: a realist evaluation

**DOI:** 10.1136/bmjqs-2025-019749

**Published:** 2026-04-20

**Authors:** Nicola Brennan, Ellie Reynolds, Tristan Price, Geoff Wong, Jennifer Cleland, Helen Lloyd, Lyndsey Withers, Thomas Gale

**Affiliations:** 1 Centre for Applied Medical Education and Healthcare Workforce Research (CAMERa), Faculty of Health, University of Plymouth, Plymouth, UK; 2 Primary Care Health Sciences, University of Oxford, Oxford, UK; 3 Lee Kong Chian School of Medicine, Nanyang Technological University, Singapore; 4 School of Psychology, University of Plymouth, Plymouth, England, UK; 5 Patient Partner, University of Plymouth, Plymouth, UK

**Keywords:** Medical education, Qualitative research, Evaluation methodology

## Abstract

**Background:**

The optimal performance of doctors is critical to delivering high-quality, safe healthcare. However, 6–12% of doctors may experience challenges that impact their work performance. In many countries, including the UK, there is variation in the practice and quality of professional support services between different types of organisations. The aims of our study were (1) to identify why, how, in what contexts and for whom professional support works, (2) to develop a guide for healthcare organisations to use to optimise professional support.

**Methods:**

We carried out a realist evaluation consistent with Realist And Meta-narrative Evidence Syntheses: Evolving Standards (RAMESES) II standards. 45 interviews were conducted with professional support staff and doctors who had undertaken professional support across seven sites in England. Interviews were analysed using a realist logic. To develop the guide, six workshops were held with the same groups plus a patient and public involvement group.

**Results:**

We identified six principles of effective professional support, based on 47 context-mechanism-outcome configurations. (1) Work-place culture influences support-seeking behaviour. (2) Trust and psychological safety are central to enable candid conversations and engagement. (3) Doctors can then develop self-awareness and situational awareness, reframe challenges, accept responsibility where appropriate and recognise structural factors underpinning their difficulties. (4) Doctors are more likely to feel motivated to engage through positive framing. (5) Personal and professional growth occurs when doctors are empowered to make changes to their practice. (6) Cultures that stigmatise help-seeking undermine support, while those that model vulnerability and normalise support enhance the likelihood of positive outcomes.

**Conclusions:**

Our guide provides step-by-step advice to identify key actions for those delivering professional support . Since the realist approach identifies principles and causal explanations, the findings are likely transferable to other settings/countries.

**WHAT IS ALREADY KNOWN ON THIS TOPIC**

- There is a great deal of variation in the practice and quality of professional support services between different types of organisation.
- There is limited evidence on what constitutes good practice.

**WHAT THIS STUDY ADDS**

- High-quality professional support services are delivered by facilitators who consistently cultivate trust, psychological safety, self/situational awareness and motivation among the doctors they support.
- Organisational cultures that model vulnerability and normalise support increase the likelihood of doctors seeking and/or engaging with professional support in the first place.

**HOW THIS STUDY MIGHT AFFECT RESEARCH, PRACTICE OR POLICY**

- The implementation of the principles and the guide could optimise professional support programmes in the UK, and in other settings/countries around the world.

## Introduction

 High-quality and safe patient care needs doctors to perform optimally.^1^ However, approximately 6–12% of doctors face challenges that impact their work performance at any one time.^2^ Doctors can experience problems at any career stage,^3^ and it is crucial that such issues are identified quickly and, where appropriate, the doctor is provided with relevant support.^4^ However, this may not be straightforward. In the UK, for example, there is variation in the practice and quality of professional support services between different types of organisation in the National Health Service (NHS),^5^ with limited evidence on what constitutes good practice. Importantly, existing literature highlights the need to develop the theoretical underpinnings of support interventions for practising doctors to help with transferability of best practice across contexts.^4 6–9^ Understanding how professional support works, for whom and in what contexts is, therefore, essential to designing effective programmes. Otherwise, professional support programmes may be ineffective, wasting doctors’ time and taxpayers’ resources, and potentially leaving patients at risk.

Before continuing, it is important to consider what we mean by professional support.^5 10^ We view ‘professional support’ as an overarching term for interventions designed to support doctors experiencing performance, behavioural, health or professional development challenges. These interventions include remediation, performance management, mentoring, coaching, supervised practice and well-being support. Professional support is aligned with an educational or developmental act,^8^ with the focus being on learning and adopting better ways of practising across individual, workplace, team and system levels.^11^ Professional support can be mandatory, but doctors may also voluntarily self-refer for professional support.

Our programme of research focuses on investigating the theoretical underpinnings of support programmes for practising doctors.^12^ Our 2021 realist review of international literature (n=141 studies) addressed how remediation interventions produce their effects (REaliSt evaluaTions of prOfessional suppoRt and rEmediation, (RESTORE) 1).^12^ It developed a programme theory of why, how and for whom remediation programmes work. We found that they are effective when a doctor’s insight and motivation are supported and developed, and behaviour change is reinforced. However, we also identified significant knowledge gaps, specifically, the absence of detailed high-quality data to test (confirm, refute or refine) the programme theory, particularly regarding contextual influences on effectiveness. Furthermore, the nature of the available literature meant that the review focused predominantly on an individualised notion of performance rather than encompassing relational, structural and systems factors.

The current study addresses these gaps via a realist evaluation of professional support programmes in the NHS in England, UK. The aim was: (1) to identify why, how, in what contexts and for whom professional support works for practising doctors; and (2) to develop a guide to optimise professional support for doctors in the NHS, which may also be useful in healthcare contexts in other countries.

## Methods

### Study design and rationale for using realist evaluation

We conducted a realist evaluation consistent with Realist And Meta-narrative Evidence Syntheses: Evolving Standards (RAMESES) II standards.^13 14^ Data were collected through semi-structured interviews with key stakeholders to explore the mechanisms underpinning professional support. This approach was used because our realist review (RESTORE 1)^12^ identified that professional support programmes are complex; how well they work (or not) depends on context, who delivers them and how they do it. Although some strategies (eg, training, placements) are commonly used in professional support, the overall professional support package is usually (or should be) tailor-made for the individual doctor, their issues, circumstances and working environment. The realist evaluation approach is particularly apt for complex problems of this nature.

### Initial programme theory

**Figure 1:**
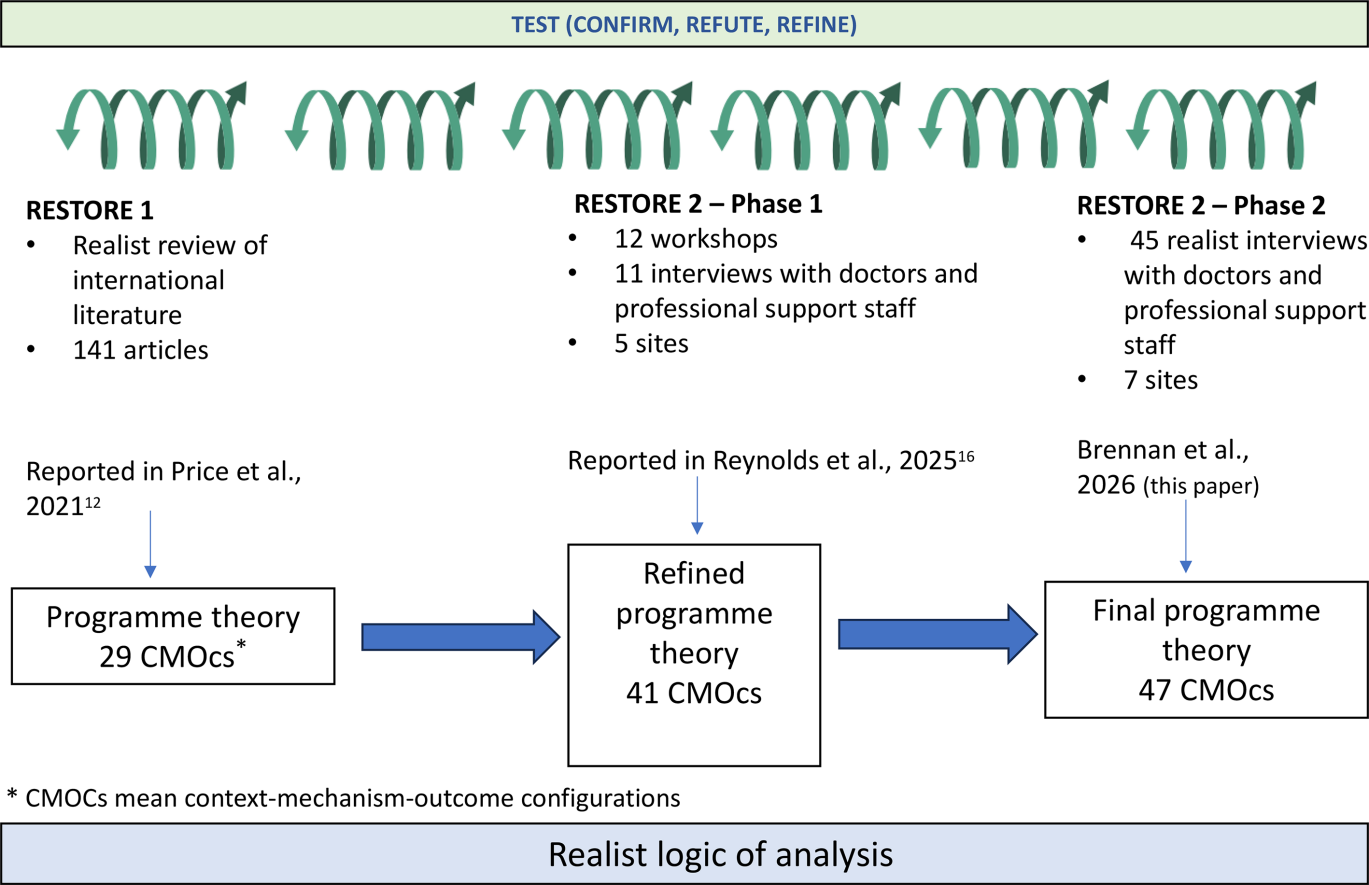
Overview of programme theory refinement developed across the multiphase programme of work.^12 16^

Our initial programme theory was developed through two separate research studies. First, we developed a programme theory through a realist review of international literature (the RESTORE 1 study).^12^ We then refined this programme theory^15^ using data collected through workshops and interviews in Phase 1 of the current research, the RESTORE 2 study.^16^ The interviews in Phase 1 were conducted with professional support staff to understand how their service worked. The interviews with doctors who had undertaken professional support focused on their experiences of professional support, their views on the importance of the RESTORE 1 recommendations and potential outcome measures of professional support. The refined programme theory consisted of 41 context–mechanism–outcome configurations (CMOcs) (online supplemental additional file 1) which we then tested using the interview data outlined in the following sections of this paper. Figure 1 provides an overview of programme theory refinement across our programme of work. This paper reports the findings of the RESTORE 2 Phase 2 research.

### Study setting

Our study setting was a national public health service, the UK’s NHS. The primary focus of our study was doctors in England, who, within the NHS, are generally employed by local Trusts—semiautonomous units that manage one or more hospitals. Further detail on the structure of professional support services is available in one of our previous papers.^16^

### Characteristics of professional support programmes

There are two necessary stages to professional support programmes. The first stage is the identification of an issue, behavioural deficit or suboptimal performance. The second stage is the development of a professional support plan that identifies the intervention to address the issue and the means of delivery of this intervention. For remediation and particular types of professional support cases, there is a third stage that involves the reassessment of performance post intervention.^4^ It may not be required for all professional support activities. Our study focuses on all types of professional support, including performance-based and other approaches, for example, supporting mental health and well-being, additional training and managing career issues.

### Sites

We intentionally selected and recruited seven diverse sites. They included four NHS Acute Trust hospitals (sites A, D, F and G), two NHS England Professional Support Units/Professional Support and Well-being Services (sites C and E) and an independent professional support organisation (site B).

The NHS sites were purposively sampled to involve specific groups of doctors including primary care, secondary care, residents, consultants, specialty, associate specialist and specialist (SAS) doctors and locally employed doctors (LED). SAS doctors are experienced doctors who hold permanent positions but are not in formal training and are not consultants. LED doctors are employed by NHS trusts on local contracts rather than the nationally agreed contracts that apply to resident or SAS doctors.

The sites were also chosen to add geographical variability to promote diversity in terms of the characteristics of the doctors undergoing professional support (eg, ethnicity, place of primary medical qualification).

### Sampling and recruitment

Site leads identified suitable professional support staff as well as doctors who had received professional support to invite to take part in the study. We invited staff with different roles in the professional support process to participate, ensuring that we captured diverse perspectives. Email invitations included a study information sheet and consent form, noting the opportunity for participants to speak to the researchers by telephone or video call to discuss the project in more detail if they wished. We also used other recruitment methods, including ‘snowball sampling’ and advertising the study via X/Twitter.

### Data collection methods

Semistructured interviews were conducted with professional support staff and doctors who had undertaken professional support at each of the sites. The interviews were conducted using a realist approach.^17^ The interview schedule focused on testing (confirming, refuting or refining) the existing programme theory with three major sections around trust and psychological safety, insight and perspective. The schedule iteratively evolved as interviews progressed and particular sections of the programme theory were further explored.

Interviews were conducted using Microsoft (MS) Teams or by telephone. All interviews were recorded using the MS Teams recording function and/or an electronic voice recorder and were transcribed by a professional transcriber. Data were anonymised and stored securely on MS OneDrive for Business.

### Data analysis

Interview transcripts were entered into NVivo V.14 (a qualitative data analysis software) for analysis (see online supplemental additional file 2 for further information on the coding process). We used the same realist logic of analysis as previously.^18 19^ This is a way of interrogating theory with data and using theory to understand patterns in data to further test the programme theory. A realist analysis of data follows a generative explanation for causation, that is, an outcome (O) of interest was generated by relevant mechanism(s) (M) being triggered only in a specific context (C). These causal explanations are provided in the form of CMOcs.^20^

We used retroductive reasoning to infer and elaborate on the mechanisms. Retroductive analyses seek to identify the hidden causal processes that lie beneath identified patterns or changes in those patterns.^21^ Thus, our approach involved repeatedly going from data to theory, to refine explanations about the occurrence of certain behaviours.

The analysis was conducted by ER in constant collaboration with NB and TP. Drafts of CMOcs were considered alongside data in full team meetings. Repeated rounds of coding and team discussion facilitated further insight and refinement.^22^

### Development of the guide

The final phase of the study was to develop a guide to optimise professional support in the NHS (and which we believe will also be useful to other healthcare contexts). We drew on: (1) our refined RESTORE 2 programme theory; (2) our experience gained through previous phases of the research; and (3) focused engagement with key stakeholders.

To develop the guide, we held virtual workshops with professional support staff at five of the study sites (sites A–D, F and G combined) and patient and public involvement (PPI) participants. During the workshops, the guide was screen-shared with participants. The workshop leads then went through the document section by section, asking participants for their feedback on clarity, relevance, format, language and tone, and whether there were any gaps. The workshops were held on MS Teams, lasted 90 min and were recorded. Participants who were unable to attend workshops either took part in 1:1 recorded interviews to the same format on MS Teams and lasting approximately 60 min, or provided input via email. The workshop and interview feedback was then used to refine the guide.

### Team reflexivity

Reflexivity was an integral part of the whole research process^23^ and we built regular opportunities for reflection and discussion into team meetings. The multidisciplinary team of experienced researchers and clinicians, with a range of topic area and methodological expertise, brought diverse perspectives (eg, social science, anthropological, psychological, educational, clinical, PPI) to the research. The medically qualified team members came from diverse specialties including general practice and anaesthesia. These aspects of diversity were important, given the scope and complexity of professional support for doctors.

### Patient and public involvement

Stakeholders were engaged in all phases of the RESTORE 2 study via a stakeholder group. This consisted of two sets of participants; PPI lay members and key stakeholders in the professional support process (eg, doctors who had undertaken professional support, those delivering professional support programmes, policymakers, coaches). The stakeholder group met regularly (six times over the lifetime of the study). They helped us to sense-check emerging findings and provided additional feedback and advice that enabled us to optimise our outputs and dissemination plans and produce feasible and practical recommendations. The stakeholder group also involved members from key organisations who will use the guide or be in a position to promote its use.

## Findings

### Characteristics of settings and participants

We conducted 45 interviews with professional support staff and doctors who had undertaken professional support across seven sites (table 1). The key characteristics of the interview participants are presented in table 2. Interviews took place between 15 September 2023 and 19 July 2024. Interviews lasted between 18–78 min with an average length of 39 min.

**Table 1:** Overview of realist interview sites and participants

Site reference	Site type	Region of England	No. of professional support staff	No. of doctors that had undertaken professional support
A	Acute Trust	South West	2	5
B	Professional support organisation	National	5	1
C	Professional support unit/professional support and well-being service	South East	4	19
D	Acute Trust	Midlands	3	1
E	Professional support unit/professional support and well-being service	South East	1	0
F	Acute Trust	South East	0	3
G	Acute Trust	South West	1	0
			16/45	29/45

**Table 2:** Key Characteristics of the 45 interview participants

Characteristic	Diversity achieved	
Doctors who had undertaken professional support (n=29) (mandated professional support n=2)	Medical career stages	Specialty trainee 72% Consultant 11% SAS* 11% GP resident† 3% Foundation‡ 3%
	Medical specialities	ICU, emergency medicine, paediatrics, anaesthetics, pathology, general medicine, primary care, surgery, neurology, gastroenterology, obstetrics and gynaecology, general practice, palliative, ophthalmology, oncology, intensive care
	Primary reason for undertaking professional support	Mental health support 38% Career support 20% Returning to work/training following health issues 20% Examination support 7% Performance 7% Assertiveness and confidence 4% Experienced bullying 4%
	Primary medical qualification	UK 86% Europe 7% Rest of World 7%
Professional support staff (n=16)	Role	Coach 25% Manager 19% Mentor 19% Director 13% Responsible officer 12% Advisor 6% Human resources 6%
Age range		25–35 31% 36–45 33% 46–55 20% 56–55 13% 56–65 3%
Gender		Female 62% (n=28) Male 38% (n=17)
Ethnicity		White 62% (n=28) Asian 31% (n=14) Black, mixed race or other 7% (n=3)

* SAS doctors—specialty, associate specialist and specialist doctors. These are experienced doctors who hold permanent positions but are not in formal training and are not consultants.

† GP—general practitioner. GPs provide general medical treatment for patients outside the hospital setting.

‡ Foundation doctor—a newly graduated doctor in the first 2 years of their postgraduate training, following medical school in the UK.

ICU, intensive care unit.

To develop the guide, we conducted six workshops with 23 participants, plus six interviews with doctors and staff. A further two participants provided feedback via email as they could not attend a workshop or interview (table 3).

**Table 3:** Overview of data collection to develop the Guide

Site	Workshop (no. of participants)	Feedback via email	Interviews with doctors	Interviews with professional support staff	Site total
A	3	2	2	1	**8**
B	3	0	0	0	**3**
C	3	0	2	0	**5**
D	5	0	0	0	**5**
G and H combined	3	0	1	0	**4**
Patient and public involvement representatives	6	0	0	0	**6**
Overall total	**23**	**2**	**5**	**1**	**31**

### Characteristics of optimal professional support

**Figure 2:**
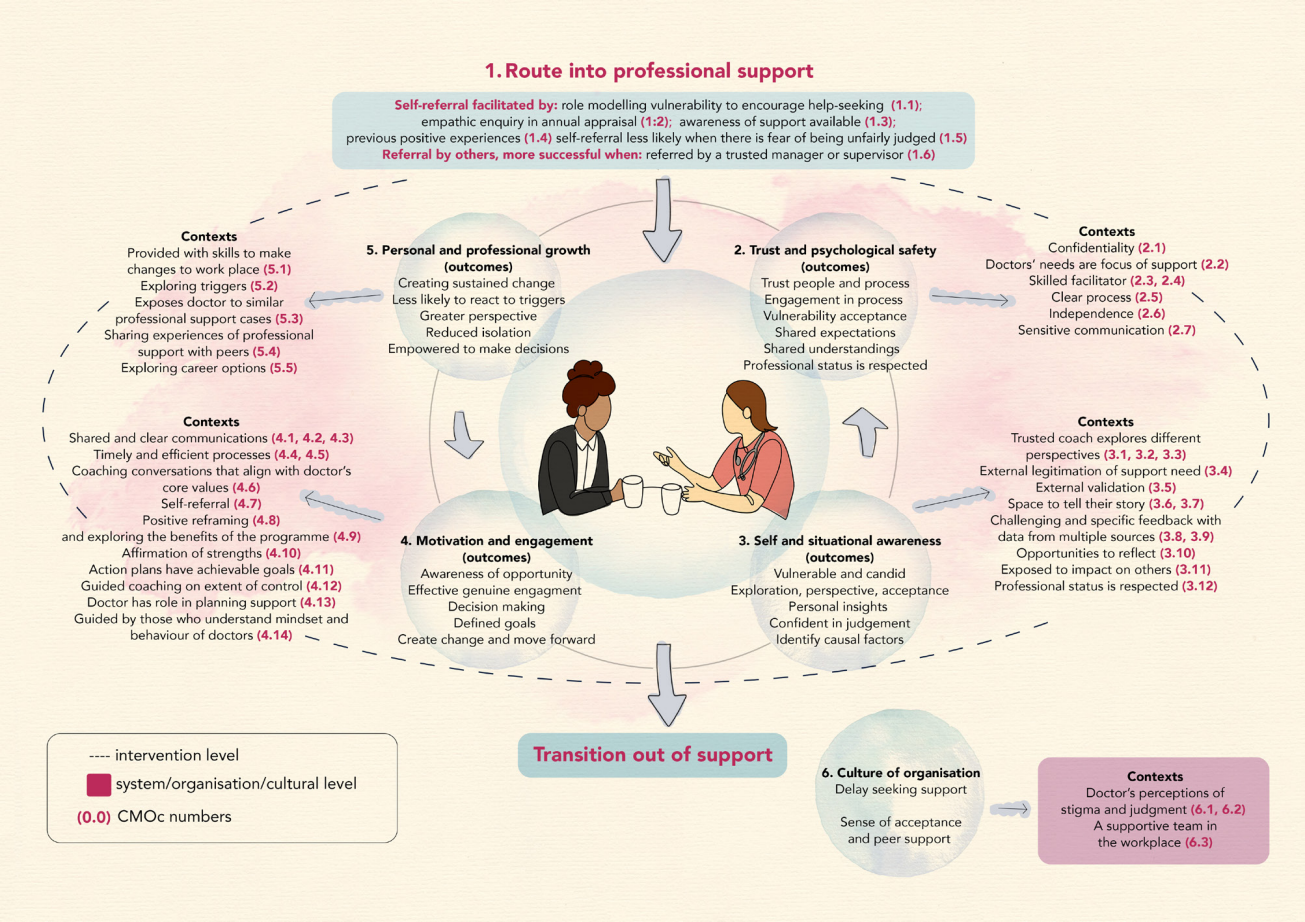
RESTORE 2 programme theory of professional support. CMOc, context–mechanism–outcome configuration.

The RESTORE 2 programme theory (figure 2) consists of six groups of outcomes as below, underpinned by 47 CMOcs (online supplemental additional file 3). See online supplemental additional file 4 for a selection of CMOcs with supporting data. Five of the outcome groups relate to the intervention, with the sixth group being concerned with broader organisational culture issues that directly impact on the function of support programmes.

#### Routes into professional support and self-referral

Workplace cultures that role model vulnerability, encourage help-seeking and have clearly explained pathways into professional support are more likely to foster environments that facilitate self-referral (CMOCs 1.1, 1.3–1.4). Annual appraisals or other performance reviews can help explore support needs and increase the likelihood that doctors can be signposted to support at an earlier stage (1.2). Mechanisms in this section of the programme theory relate to agency (control over a process) and social support (resources from networks that guide behaviour and influence decisions).

#### Building trust through psychological safety

Trust in both the professional support process and the people involved is essential to fostering meaningful conversations around support needs. Programmes can foster trust by being candid and clear about timelines and expectations (2.5), and by promoting a culture of dignity and confidentiality (2.1, 2.4, 2.7). Doctors need a safe space to discuss the challenges that they are experiencing, and these challenges should be the focus of early conversations within professional support programmes (2.2). Mechanisms that develop these trusting relationships revolve around psychological safety, which can help a doctor express and embrace their vulnerability and facilitate the conversations that will underpin the rest of the process. Important proximal outcomes to trust in the early stages of a process of support involve developing a shared understanding of a given situation, and shared expectations of the support process.

#### Self-awareness and situational awareness

Support and remediation programmes work when they help those receiving support to develop self-awareness and situational awareness. When relationships with facilitators are built on trust, and doctors are given space to share their story, cognitive reframing can occur, developing insights into the doctor’s responsibilities and accepting which aspects of a situation they have control over, and which they do not (3.1, 3.2, 3.6). Mechanisms here involve reflection and cognitive reframing that prompt re-evaluation.

An important outcome within this category is acceptance. This can involve a doctor accepting the need for change and then taking responsibility, which is achieved by a supportive facilitator delivering challenging feedback who encourages engagement with the perspective of others’ (3.3, 3.8, 3.9). Outcomes at this stage may include a shared understanding that issues stem from structural, cultural or team-related factors (3.7, 3.10), and conversations may focus more on navigating these rather than trying to change the individual doctor. Or it may be a mixture of both.

#### Engagement and motivation

Doctors are more likely to engage when they know what to expect on their professional support journey (4.1, 4.2, 4.4, 4.5), when there is clear communication about developments along the way (4.3) and when they feel in control and believe that outcomes are achievable (4.11–4.13). Programmes foster this through efficient, focused processes and by developing plans *with* the doctor.

Remediation and professional support programmes are often most effective when a doctor self-refers, as they feel greater control from the beginning (CMOC 4.7). Doctors who have been referred into professional support programmes may not agree with their referral, but a skilled facilitator may still be able to support a doctor to recognise the benefits of engaging with aspects of the programme as an opportunity for development (4.9).

#### Personal and professional growth

Personal and professional growth occurs when doctors are empowered to make changes to their practice and are supported with the skills to speak up and address workplace issues that have led to professional support needs (5.1). If relevant to a particular issue, it can be helpful when a doctor is supported to recognise their personal emotional triggers and develop strategies for dealing with them (5.2). If a doctor has an opportunity to share their experience or hear about others in a similar situation, this reduces isolation and shame and provides greater perspective (5.3, 5.4).

#### Organisational culture

There are broader structural and cultural factors that impact on whether a process of professional support is effective. Workplace team cultures can affect whether a doctor is likely to share their difficulties and exhibit a readiness to seek help. Some organisational cultures foster shame around seeking support, especially if it involves taking time out of practice, and this can discourage help-seeking (6.1–6.3). Workplace cultures that actively aim to normalise seeking support and remove the stigma of undergoing professional support are likely to contribute to a successful process of support.

### The guide

The guide contains detailed recommendations on how to optimise professional support services for doctors (online supplemental additional file 5). It is primarily aimed at the setting in which we collected data, the NHS in England, UK, but is likely to be broadly applicable to many different healthcare environments. It is centred on the experience of doctors in professional support and follows the ‘pathway’ of professional support, spanning from the initial point of a doctor realising they may need help, to the point where the doctor can work without their performance being affected.

## Discussion

The aim of this research was to: (1) identify why, how, in what contexts and for whom professional support works for practising doctors; and (2) develop a guide to optimise professional support for doctors in the NHS. Our study found that professional support programmes for doctors are most effective when embedded in a culture that normalises help-seeking and fosters trust and psychological safety. Self-referral routes work best as they promote agency from the outset. Trust and psychological safety are central to engagement, allowing doctors to develop self-awareness and situational awareness, reframe challenges, accept responsibility where appropriate and recognise structural or cultural factors underpinning their difficulties.

Skilled facilitators are essential, combining supportive challenge with openness to multiple perspectives and helping doctors explore emotional triggers. Through collaborative planning and reflection, facilitators foster agency and motivation. Well-structured programmes support both personal and professional growth, enabling doctors to adapt their practice, manage triggers and address workplace factors affecting performance and well-being.

Cultures that stigmatise help-seeking or equate it with failure undermine support, while those that model vulnerability and normalise support enhance the likelihood of positive outcomes.

The primary data from this study both support and extend the RESTORE 1 programme theory developed in our earlier realist review.^12^ While the current study (RESTORE 2) broadly aligns with the earlier programme theory, it introduces several refinements. By engaging directly with lived experience, this study has clarified the specific processes and structures within healthcare systems that result in the delivery of effective professional support. Notably, it highlights the importance of programme entry routes, distinguishes trust from psychological safety as precursors to insight and differentiates self-awareness from situational awareness. By focusing on professional support in RESTORE 2 (rather than the more limited scope of remediation in RESTORE 1), we moved beyond the assumption that outcomes must involve ‘behaviour change’, to a focus on personal and professional growth. The refined programme theory is also broader, encompassing cultural and systemic factors.

This study adds to the broader remediation and professional identity literature by highlighting trust and psychological safety as core elements of educational and support systems in healthcare.^24^ These principles may apply more broadly but have particular relevance in postgraduate medical education, where engagement in support programmes is tightly linked to professionalism.^24^ Loss of autonomy—especially in mandated professional support—can challenge doctors’ professional identity.^25^ The concept of psychological safety is well established in education literature, but our findings clarify its practical application. Psychological safety exists when doctors can openly confront distressing issues, though such spaces inevitably involve negative emotions. Recent research shows emotions are not incidental but integral to professional development.^26–28^ Nascent research on the role of emotions in learning suggests that insights from other fields may suggest ways to harness emotion to improve learning in medical education more broadly, and professional support more specifically.^26 28^ In practice then, psychological safety is best understood as a core underpinning process that enables insight at key points. Growth does not depend on the absence of negative emotion but on learning to recognise and navigate it.

Our programme theory also connects with wider healthcare literature, particularly the person-centred coordinated care (P3C) model.^29^ Both programme theories emphasise early conversations, a shared understanding of the problem, individualised support, trust-building, awareness and agency. The analogy is clear: doctors in professional support occupy a position similar to patients navigating systems of care. Indeed, prior research in UK medical schools has shown that doctors often frame remediation experiences in ‘patient narrative’ terms.^30^ Both the P3C model and our realist programme theory of professional support stress agency, skilled facilitation and trust as central to effective support,^29^ and these factors are likely to be relevant to professional support in all healthcare systems.

### Strengths and limitations

A strength of this research relates to the use of a realist approach which successfully accommodated the complexity of the topic across multiple settings with broad sampling.

The research team’s and stakeholder group’s different disciplinary/professional backgrounds brought different perspectives to the research.

A potential weakness relates to the number of participants and their diversity in terms of the doctors’ reasons for undertaking professional support. We had planned to interview 75 participants but ended up with 45. This was despite repeated recruitment drives using different strategies over a long time frame (12 months). The low recruitment of doctors may have been related to the sensitivity of the topic and doctors’ reluctance to speak about their experiences. The low recruitment of staff may have been due to their busy schedules and because the professional support teams at the sites were smaller than anticipated. This is a potential issue for others to consider when carrying out research in this space.

Recruiting doctors who had undertaken mandated professional support was challenging. However, this is counterbalanced by the fact that many of the staff that were interviewed had provided support for doctors undertaking mandated professional support and were thus able to provide perspective on that situation.

Although we sampled for diversity, the seven sites were self-selecting and thus may not be representative of all Trusts in England. Our participants were not representative of the balance of doctors working in the UK in terms of place of primary medical qualification (PMQ) (UK or overseas) .

### Implications for policy and practice

This study demonstrates that professional support for doctors is most effective when framed broadly as a developmental resource rather than narrowly as remediation. Organisations should design services that emphasise professional support rather than deficit-focused interventions, reducing stigma and encouraging earlier engagement.

A central implication of this is the importance of routes into support. Systems should enable and normalise self-referral, while also ensuring clear pathways for those who may be too unwell or uncertain to initiate support themselves.

Equity of access is another key consideration. Support should be available to all doctors, regardless of training status or employment contract, with particular attention to the needs of international medical graduates (IMGs), who may face disproportionate challenges.^31^

Finally, organisational culture is pivotal. Cultures that stigmatise help-seeking undermine support, whereas those that role model vulnerability and embed professional support into the fabric of practice promote agency and professional identity. As part of this study, we developed an animation which aims to normalise professional support and promote cultural change: https://www.youtube.com/watch?v=Xo-k_e08V_I

For healthcare systems internationally, the implication is clear: effective support requires both well-designed and delivered interventions, and cultures that legitimise and value help-seeking as integral to professional life.

### Implications for future research

Realist approaches are particularly well suited to this complex topic. Future research could test our programme theory in other countries, or other professions, for example, dentistry, nursing and midwifery, or pharmacy, or with other populations, for example, IMGs, primary care/general practitioners/community doctors.

We noted that a high proportion of those receiving support were doctors in training (residents). We are not aware of any statistics on the medical career stages of doctors that have undertaken professional support. However, we suspect that residents may be better at asking for support as they are in training and they view support as part of their training. There may also be generational differences in help seeking and differences in access to support services depending on seniority or employment status. These possibilities merit further investigation.

While the data were collected in one of the UK’s four constituent countries, we anticipate that the principles and causal explanations could be relevant in other settings and countries. This merits further research, particularly in countries and health systems with diverse contexts and cultures, and potentially different attitudes towards underperformance and support.

## Conclusion

High-quality professional support for doctors is possible, but it requires well-designed services that are consistently provided by facilitators who are skilled in developing trust and psychological safety, self-awareness and situational awareness and motivation in the doctors they support. Organisational cultures that model vulnerability and normalise support increase the likelihood of doctors seeking and/or engaging with professional support in the first place. Our guide provides step-by-step advice for NHS organisations to do this. The findings are likely to be transferable to other settings and countries, since using a realist approach led to the identification of broadly applicable principles and causal explanations.

## Supplementary material

10.1136/bmjqs-2025-019749 online supplemental file 1

10.1136/bmjqs-2025-019749 online supplemental file 2

10.1136/bmjqs-2025-019749 online supplemental file 3

10.1136/bmjqs-2025-019749 online supplemental file 4

10.1136/bmjqs-2025-019749 online supplemental file 5

## Data Availability

No data are available.
