# Supplementary material for: Optimising professional support for doctors who experience work performance issues: a realist evaluation
Source: BMJ Qual Saf. 2026 Apr 20;35(8):e019749. doi: 10.1136/bmjqs-2025-019749 (PMC13479272; doi:10.1136/bmjqs-2025-019749)
Supplement: Supplementary file 1 [file bmjqs-35-8-s001.pdf]

**RESTORE 2 Phase 1 Context-Mechanism-Outcome Configurations (CMOcs)**		
**Psychological safety, relationships built on trust, candour and vulnerability**		
**a. Psychological safety**		
1	1.1	When a doctor fears the consequences of professional support or does not trust the professional support process (C), an environment of trust will not develop (O) because they do not feel psychologically safe (M). An intervention strategy that can be used to change this context is the provision of a safe space where issues can be discussed in confidence.
2	1.2	When a doctor feels that their discussions are confidential and is able to express any negative emotions they feel (C), they will be more likely to feel psychologically safe (M), leading to an environment of trust (O) and a readiness to explore perceptions of their performance (O)
3	1.3	When the doctor's needs are the focus of professional support (rather than process) trust is more likely to develop (O) because the doctor feels psychologically safe (M)
4	1.4	If a doctor accepts that there is a performance issue but does not receive validation of their professional motives/unconditional positive regard/affirmation of their professional identity (C) then identity dissonance (M) may lead to rejection of medical professional identity (O)
**b. Trust**		
5	1.5	When the doctor is able to build up a relationship with a facilitator over a period of time (C), the doctor is more trusting of the facilitator (M) and the facilitator can tailor their support to their knowledge and understanding of the doctor's needs (M) leading to a (O)
6	1.6	When the process of supporting a doctor through professional support is guided by someone from outside the doctor's employing organisation (C), then the process will be perceived as less threatening (M), leading to more meaningful engagement (O) and trust in the process (O)
7	1.7	When the doctor has been referred to the service by their educational supervisor (C) they are less likely to trust the service or staff as non-judgmental advocates (M) leading them to be less likely to engage (O)
8	1.8	When the doctor has referred themselves to the service (C) they are more likely to feel in control of the process (M) and trust those who they work with (M) leading to a greater likelihood of engagement (O)
c. **Managing expectations (safety)**		
9	1.9	When a doctor knows what to expect from the process of professional support, and is aware of what is expected from them (C), they will be more likely to feel psychologically safe (M), leading to an environment of trust (O) and a readiness to explore perceptions of their performance (O)
d. **Candour and vulnerability**		
10	1.10	The existence of a confidential, non-judgmental, safe space where the doctor is held in unconditional positive regard (C) leads to the doctor feeling safe (M) being vulnerable and candid with the facilitator (O)

**Gaining insight and perspective**		
**a. Insight**		
11	2.1	When doctors trust their coaches (C), challenging feedback delivered in a supportive manner (C) can invoke insight (M) leading to an acceptance of the need to change (O)
12	2.2	When conversations are focused on the implications and consequences of a doctor's actions (C), by a supportive and independent coach (C) insight is more likely to develop (O) because the doctor reflects on practice (M)
13	2.3	When a doctor gets feedback on their engagement with the professional support process (C), they are more likely to accept the feedback (O) because it is happening in real time
14	2.4	When the process highlights the impact on others (C), the doctor is more likely to engage (O) because they have empathy for others as part of their professional identity (M)
15	2.5	When coaches move conversations onto the future (C), doctors are more likely to engage (O) because they can see a resolution (M) and imagine a future post professional support (M)
16	2.6	When feedback contains specific performance data and/or clear examples of reported behaviours, and is derived from a number of different sources (C), it is more likely to be acceptable the doctor (M), leading to insight and self-awareness (O)
17	2.7	When feedback is perceived as a generalised judgement about an individual doctor, the doctor is more likely to be defensive (C) and therefore go into denial (M), leading to rejection of the feedback (O) and/or rejection of the standards to which that feedback pertains (O)
**b. Perspective**		
18	2.8	When the doctor is able to explore with a coach those aspects of their work relationships or environment that have caused problems (C) then they gain perspective on the situation they are in and realise that they are not the source of the problem (M), leading to greater motivation to engage (O)
19	2.9	If a coach or mentor is able affirm a doctor's strengths and offer perspective (C) then the doctor is more likely to accept negative feedback (O) because they have received professional affirmation (M)
20	2.10	When a coach is able to guide the conversation to a discussion of core values (C), then perspective, i.e. "this is what is really important to me" (M) will facilitate motivation (O)
21	2.11	When a doctor explores their own emotional triggers (C) then they are less likely to react to these triggers (O) because they are self-aware (M)
22	2.12	If feedback data are presented in a way that makes the problem seem manageable (C) then perspective (M) may lead to the doctor accepting the need to change performance or behaviours (O).
23	2.13	A doctor's professional identity may feel less threatened (O) when careful and supportive coaching allows them to explore a situation objectively (C), so they gain perspective (M)
**Motivation**		
**a. Agency and control**		

24	3.1	When an individual self refers for support, they are more likely to engage (M) because they have agency (C) and because there is less stigma (C)
25	3.2	When the doctor is able to identify their own development and learning needs, (C) then they have more perceived control over the process (M), leading to greater motivation to engage (O)
26	3.3	When a doctor is given specific strategies for learning or behaving (C) then, because of improved self-efficacy (M) they have greater motivation (O)
**b. Positive reframing**		
27	3.4	When professional support is framed as part of professional development (C), doctors may undergo cognitive reframing of experience (M), increasing motivation and creating a sense of opportunity (O)
28	3.5	When a doctor receives empathic support from a coach or from peers which includes a positive reframing of the support process (C), it increases their sense of self-worth (M) and gives them a sense of belonging (O)
29	3.6	When a doctor can share their experience with others in a similar position or with similar challenges (C), then a sense of belonging (M) can reduce isolation and increase motivation (O)
**c. Value-congruence**		
30	3.7	When a doctor is coached to explore values (C), goals are meaningful and congruent with values and ambitions (M), increasing motivation (O)
31	3.8	When a doctor undergoing support can be coached to visualise realistic and achievable outcomes (C), they may develop more motivation to achieve these goals (O) because of the value congruence between goals and ambitions (M). An iterative process of goal exploration may help to achieve this.
**d. Managing expectation (process)**		
32	3.9	When the process of support sensitively explores career options including the possibility of leaving medicine (C), doctors feel more emotionally prepared for this possibility (M) and feel less threatened (O)
33	3.10	When doctors know what to expect in the process of professional support (C), they are more likely to stay engaged in the process (O) because they feel in control (M) understand the process as a necessary part of their development (M)
34	3.11	When a doctor understands the consequences of not changing their behaviour or improving performance (C), then they may be able to evaluate the costs and benefits of change (M), and may be motivated to engage with professional support (O) or change their goal (O)
**e. Zone of proximal development**		
35	3.12	When an interactive process of coaching explores experiences and struggles in depth (C), the realisation of new possibilities (M) may lead to the identification of outcomes that go beyond the initial reason for engagement in the service (O)
**Personal and professional development**		
**a. Reflection**		

36	4.1	When the professional support process includes opportunities for doctors to informally reflect on their ongoing engagement with the process (C), they have the opportunity to integrate the insights gained (M), leading to new insights and greater perspective (O)
37	4.2	When a doctor has been guided through what feedback means (C), then they are more likely to engage with the feedback (O) because it makes sense to them (M). Intervention strategies that may help to bring this about include regular face-to-face meetings and open reflective questioning from a trained coach.
38	4.3	When feedback makes sense to a doctor (C), dissonance is more likely to be invoked (M), leading to the doctor gaining further insights into their performance or behaviours (O)
**b. Practicing skills**		
39	4.4	When educational supervisors and workplaces are made aware of what support a doctor requires in practice (C) and are willing to support the doctor (C), then positive improvements are more likely (O) because the doctor is supported to integrate new knowledge and experiences into their learning (M)
**Emotional availability of facilitators**		
**a. Emotional support**		
40	5.1	When a coach or member of professional support staff is provided with the emotional support they need to carry out their role (C), they are more able to provide empathic support (M) to the doctor who is turn feels safer (O) and engages more with the process (O)
41	5.2	When a coach or member of professional support staff is provided with the support they need to carry out their role (C), they are more likely to stay emotionally engaged in their role (M) leading to them remaining in their role long enough to develop organisational memory (O) and build relationships with other staff and clients (O)
