# Supplementary material for: Optimising professional support for doctors who experience work performance issues: a realist evaluation
Source: BMJ Qual Saf. 2026 Apr 20;35(8):e019749. doi: 10.1136/bmjqs-2025-019749 (PMC13479272; doi:10.1136/bmjqs-2025-019749)
Supplement: Supplementary file 2 [file bmjqs-35-8-s002.pdf]

Additional file 2 – Data coding and refining CMOcs across programme of research

1. Data coding

Interview and workshop transcripts collected as part of RESTORE 2 phase 1 were entered into NVivo 14 (a qualitative data analysis software) for analysis and initially thematic coded thematically to get a broad overview of the data set. The next step of our coding process involved listing each of the RESTORE 1 context-mechanism-outcome configurations (CMOcs) as a code in NVivo and coding any relevant sections of data from RESTORE 2 phase 1 (see Figure 1) that we judged to be relevant to each code in order to confirm, refine or refute each of the existing CMOcs. We also added new CMOcs that we identified in the data and discarded some CMOcs that were not supported by the data. This resulted in 41 CMOCs (Additional file 1)

We then repeated the process with the interview data collected in RESTORE 2 Phase 2. The interview transcripts were uploaded into NVivo. The 41 CMOcs were listed as codes. The data collected in RESTORE 2 Phase 2 were then coded to the updated CMOcs. After becoming more familiar with the data and dominant themes that were being repeated in the interviews, but which weren't covered by the existing CMOcs, more CMOcs were developed to account for the data we had. This resulted in our final set of 47 CMOcs. For an example of how a CMOc was refined using data from the different phases of the study see Additional file 2.

Where possible and relevant other variables (e.g. the characteristics of the doctors and the settings they work in) were entered into Excel for analysis and used to test any of the inferences we had made in our CMOcs.

2. Refining CMOcs across our programme of research

The following section provides an example of how a single CMOc was refined using data collected over different phases of the programme of research

RESTORE 1 CMOc 7

When a remediating doctor accepts that their perceptions of their performance or behaviours are not the same as their actual performance or behaviours (C), dissonance (M) leads to an acceptance of the need to change (O).

RESTORE 2 Phase 1 refining data

"I suppose really to say that, erm, the coaching, with coaching it, it, it's actually, well you hope it to be quite challenging in a way because it's through challenge that you get people to have, er, insight on how to move forwards. So, whilst there is that reflective aspect, erm, I suppose often on coaching you don't dwell on the reflective for long, it's how does it bring us forward." (E-WKSP-1)

"There's a presenting issue and then there is something that then gently with trust and a sense of safety and compassion and interest then, then the real thing will come to emerge." (E-WKSP-1)

RESTORE 2 Phase 1 CMOc 11

When doctors trust their coaches (C), challenging feedback delivered in a supportive manner (C) can invoke insight (M) leading to an acceptance of the need to change (O).

RESTORE 2 Phase 2 refining data

I assume it was just because he was a good counsellor, I felt comfortable talking to him, he was very good at challenging assertions, so if you said well something's like this because da, da, da, and then to go and, because this is what I did da, da, da, he would go back and say well is it like that, really explain why do you think it's like that, explain that to me, so he was very effective in his role. (Doctor)

But I think it's a refined art as well and comes with practice in terms of actually, it depends on the circumstances but in some interactions it's right to challenge them, and it's finding that balance in terms of actually by gently challenging at this point it's going benefit and open up,

and then in other interactions it's actually perceiving that actually it's time to give them space, give them more space. (Professional Support Staff)

Yeah absolutely, they didn't agree with everything I said, they did challenge and made me think about other scenarios that could not come into my mind (Professional Support Staff)

So I think if a [doctor] sort of doesn't have insight, I know that's quite a broad term, but I think just sort of explaining it from the other perspective and sort of saying that you know it could be perceived, their behaviour or actions or their performance could be perceived as you know, I'm trying to think of an example, rather than saying you know this is poor practice, I think it's just sort of explaining it from the other perspective and sort of how their actions could be perceived by others. And sometimes that sort of is just a bit softer than saying, and I think that does often work well actually, sometimes being too direct is sort of counterproductive in these situations. (Professional Support Staff)

RESTORE 2 Phase 2 CMOc 21

When doctors are given challenging feedback in a supportive manner by a facilitator they trust (C), a process of reflective reasoning (M) facilitates a doctor's acceptance of others' perspectives (O).
