# Supplementary material for: Optimising professional support for doctors who experience work performance issues: a realist evaluation
Source: BMJ Qual Saf. 2026 Apr 20;35(8):e019749. doi: 10.1136/bmjqs-2025-019749 (PMC13479272; doi:10.1136/bmjqs-2025-019749)
Supplement: Supplementary file 3 [file bmjqs-35-8-s003.pdf]

**Supplementary File 2 – List of RESTORE 2 Phase 2 CMOcs (n=47)**

| Context-Mechanism-Outcome Configurations (CMOcs) | | |
|---|---|---|
| Section 1: Routes into professional support | | |
| 1 | 1.1 | *When senior doctors' role-model vulnerability and seeking support (C), doctors are less likely to feel ashamed about their need for support (M) and are then more likely to seek support themselves (O).* |
| 2 | 1.2 | *When doctors receive genuine and empathic enquiry as part of their appraisal (C), they are more likely to be willing to disclose difficulties to a member of the medical education team (M), leading to support being provided in a timely manner (O).* |
| 3 | 1.3 | *When doctors are aware of what support services are available to them (C), and what needs the services cater for (C), they are better able to make a judgement about the most appropriate service (M) and are more likely to access the most appropriate service (O).* |
| 4 | 1.4 | *When a doctor has had a previous positive experience with a support service, or has heard positive information (C), they are more likely to perceive that service as being able to support them in a fair, non-judgmental manner (M), and so will be more likely to approach it (O).* |
| 5 | 1.5 | *When a doctor feels they will be unfairly judged by the people who are assessing them (C), because they are concerned they will not be treated fairly (M), they are less likely to self-refer (O).* |
| 6 | 1.6 | *When a doctor who is too unwell or unable to self-refer, but is referred by someone they trust, such as a manager, supervisor or training programme director (TPD) (C), they are more likely to welcome the referral (M) and engage with the professional support (O).* |
| Section 2: Trust and psychological safety | | |
| 7 | 2.1 | *When a doctor feels that their discussions are confidential (C), psychological safety (M) increases the trust between the doctor and the facilitator (O) and increases the doctor's trust in the process (O).* |
| 8 | 2.2 | *When the doctor's needs are the focus of professional support (C), the doctor will feel psychologically safe (M), and trust is more likely to develop (O).* |
| 9 | 2.3 | *When a service or facilitator is responsive and flexible (C), the doctor feels their needs are being centred in the process (M) and are more likely to trust the facilitator and open up (O).* |
| 10 | 2.4 | *When a non-judgemental facilitator takes the time to be present and really listen to a doctor (C), the doctor feels heard and valued (M) and is more likely to be willing to explore their difficulties (O).* |

| | | |
|---|---|---|
| 11 | 2.5 | *When the timetable for what needs to be done and by when is clear to doctors and supervisors (C), they will know what is expected of them (M), creating a shared understanding for both parties (O).* |
| 12 | 2.6 | *When the process of supporting a doctor through professional support is guided by someone the doctor believes to be independent of their employing organisation (C), then the process may be perceived as less threatening (M), leading to trust in the process and more meaningful engagement (O).* |
| 13 | 2.7 | *When managers approach performance conversations sensitively (C) the doctor feels heard (M) and in control (M) meaning they feel their professional status is respected (O).* |
| Section 3: Self and situational awareness | | |
| 14 | 3.1 | *When a doctor trusts the support process and trusts the support team (C), they will feel psychologically safe (M) and so willing to be vulnerable and candid with their facilitator (O).* |
| 15 | 3.2 | *When the doctor is able to explore an issue from different perspectives with a trusted coach (C) a process of reflective reasoning (M) leads to greater situational awareness and perspective (O).* |
| 16 | 3.3 | *When doctors enter professional support feeling misunderstood or unfairly blamed (C) and facilitators build a trusting, non-judgmental relationship while offering alternative perspectives (C) this can trigger reflective reasoning ("I can see how others might view it differently") (M) leading to increased openness to alternative viewpoints (O).* |
| 17 | 3.4 | *When a professional who is objective and independent affirms that the doctor has legitimate support needs (C) this external validation (M) leads to acceptance of the need for support (O).* |
| 18 | 3.5 | *When a doctor has external independent validation of their concerns (C) because they feel reassured (M) they have a renewed confidence in their own judgement (O).* |
| 19 | 3.6 | *When doctors are asked to structure their story as a narrative when sharing experiences with a facilitator (C), emotional processing (M) creates the emotional capacity to explore a problem (O).* |
| 20 | 3.7 | *When a doctor has the emotional capacity to explore a problem (C) a process of reflection and reframing (M) will assist the doctor to identify causal factors which may have contributed to the situation (O).* |
| 21 | 3.8 | *When doctors are given challenging feedback in a supportive manner by a facilitator they trust (C), a process of reflective reasoning (M) facilitates a doctor's acceptance of others' perspectives (O).* |

| | | |
|---|---|---|
| 22 | 3.9 | *When feedback contains specific performance data and/or clear examples of reported behaviours and is derived from multiple different sources (C), it is more likely to be perceived by the doctor as credible (M), leading to insight and self-awareness (O).* |
| 23 | 3.10 | *When the doctor has the opportunity to reflect between sessions (C), then the integration of the insights gained (M) facilitate self and situational awareness (O).* |
| 24 | 3.11 | *When the process highlights the impact on others (C), because it invokes empathy for others (M), doctors are more likely to be open to different perspectives (O).* |
| 25 | 3.12 | *When a doctor feels their professional status is respected (C) they are less threatened (M) and more willing to explore what has gone wrong and their role (O).* |
| Section 4: Motivation and engagement | | |
| 26 | 4.1 | *When the employing organisation actively and positively supports and updates doctors for whom professional support is being provided (C), the doctor feels included in the process and valued (M), leading to them being more likely to stay engaged in the professional support process (O).* |
| 27 | 4.2 | *When doctors know what to expect in the process of professional support (C), they feel in control (M) and understand the process as a necessary part of their development (M), they are then more likely to stay engaged in the process (O).* |
| 28 | 4.3 | *When supporting organisations and supervisors/advisors have clear lines of communication for sharing information (C) then there is a shared understanding of the problem (M), leading to the doctor being more able to engage effectively with the service (O).* |
| 29 | 4.4 | *When the doctor's employing organisation takes responsibility for ensuring the process and actions are swift (C), timely (C) and clearly communicated to the doctor (C), the doctor is more likely to feel that resolution is achievable (M) leading to a greater motivation to continue to engage (O).* |
| 30 | 4.5 | *When a doctor hears back quickly from a service and is offered a timely discussion or appointment (C), they feel their concerns are being taken seriously (M), and are more likely to feel motivated to continue to engage with the service (O).* |
| 31 | 4.6 | *When a facilitator is able to guide the conversation to a discussion of the doctor's core values i.e. "this is what is really important to me" (C),* |

| | | |
|---|---|---|
| | | *because values align (M), it expands self-awareness and increases motivation (O).* |
| 32 | 4.7 | *When the doctor has referred themselves to a service they have chosen (C), they are more likely to feel in control of the process (agency) (M) leading to a greater likelihood of engagement (O).* |
| 33 | 4.8 | *When professional support is framed as part of professional development (C), doctors may undergo positive reframing of their experience (M), increasing motivation (O), and creating a sense of opportunity (O).* |
| 34 | 4.9 | *If a doctor does not agree with their referral onto a support a programme (C) but a skilled facilitator can help them see the professional benefits of engaging with the programme (C) this can trigger the internal rationalisation ("I'm here now so I might as well make the most of it") (M), leading to genuine engagement (even if they continue to disagree with their referral) (O).* |
| 35 | 4.10 | *If a trusted facilitator or mentor affirms a doctor's strengths and offers perspective (C), increased self-efficacy (M) leads to greater motivation (O).* |
| 36 | 4.11 | *When an action plan has clearly defined and achievable goals (C), leading to an increased sense that the action plan is achievable and that they are in control of the support process (M), a doctor is more likely to feel they have something to work towards (O).* |
| 37 | 4.12 | *When a facilitator helps a doctor realise what they do and do not have control over in their professional life (C), the doctor is more able to focus on the agency they do have (M), motivating them to create change and move forward (O).* |
| 38 | 4.13 | *If the doctor has a role in planning aspects of the professional support process (C), then they may perceive that they have some control over the process (perceived agency) (M) and be intrinsically motivated to change (O).* |
| 39 | 4.14 | *When a facilitator understands the mindset and behaviour of doctors' (C), a doctor is more likely to feel understood (M) and is more likely to engage (O).* |
| Section 5: Personal and professional growth | | |
| 40 | 5.1 | *When doctors develop the skills they need to speak up or make changes in the workplace (e.g. through professional support) (C), because they feel more confident and empowered (M), then sustained positive change in the workplace is more likely (O).* |
| 41 | 5.2 | *When a doctor explores their own emotional triggers with a facilitator and develops the ability to recognise these triggers (C), they may become* |

| | | |
|---|---|---|
| | | *more self-aware (M) and less likely to react to those triggers in the future (O).* |
| 42 | 5.3 | *When the facilitator exposes the doctor to similar examples of where professional support has been required (C) and / or helps them understand that these experiences are common (C), a process of normalisation (M) results in greater perspective (O) and less shame (O).* |
| 43 | 5.4 | *When a doctor can share their experience of professional support with others in a similar position or with similar challenges (C), a sense of camaraderie (M) can reduce isolation (O).* |
| 44 | 5.5 | *When the process of support sensitively explores career options including the possibility of leaving medicine (C), doctors feel more emotionally prepared for and less threatened by this possibility (M) and more likely to be empowered to make such decisions (O).* |
| Section 6: Culture of organisation | | |
| 45 | 6.1 | *When the doctor perceives that taking time off will be judged as being 'lazy' (C), they may feel guilty (M) and fear the associated stigma (M), and delay seeking support (O).* |
| 46 | 6.2 | *When a doctor believes that seeking certain forms of help is considered a sign of weakness (C) because they see it as shameful (M), they are less likely to seek support at an early stage of difficulty (O).* |
| 47 | 6.3 | *When a doctor works in a supportive team (C), they may feel safe to share their difficulties (M) leading to a sense of acceptance and ongoing peer support (O).* |
