# Supplementary material for: Optimising professional support for doctors who experience work performance issues: a realist evaluation
Source: BMJ Qual Saf. 2026 Apr 20;35(8):e019749. doi: 10.1136/bmjqs-2025-019749 (PMC13479272; doi:10.1136/bmjqs-2025-019749)
Supplement: Supplementary file 4 [file bmjqs-35-8-s004.pdf]

Additional File 3 – Selection of RESTORE 2 Stage 2 CMOcs with supporting data

Section 1 Routes into professional support		
1	1.1	***When senior doctors' role-model vulnerability and seeking support (C), doctors are less likely to feel ashamed about the need to seek support (M) and are then more likely to seek support themselves (O).***
		I think that's really strong leadership when you [...] show vulnerability, because it then enabled everybody else to speak up about how they were feeling. (Doctor)<br><br>I think that's really important and having people who are more senior than you, and people that you look up to saying not just this is a service you can go to but actually oh actually this has happened to me and this is what I did. That is a huge change, cos that's somebody who's not only your peer but who you look up to and see as successful, and who has been in your place. So I think that definitely reduces the stigma and encourages you and shows you the positives of turning to, getting this kind of support. (Doctor)<br><br>Yeah. But I've sort of taken it that if I can, change of culture starts with good role-modelling, so if I can start to role-model and get the more senior you are the more important role-modelling is, so it trades down. (Doctor)
Section 2: Trust and psychological safety		
7	2.1	***When a doctor feels that their discussions are confidential (C), psychological safety (M) increases the trust between the doctor and the facilitator (O) and increases the doctor's trust in the process (O).***
		It's very important the [doctor] does feel comfortable and isn't concerned that things are going to be shared […] without their express consent, so I always sort of emphasise that it's a confidential discussion between us just so that, well to inform me really and see things from their perspective before sort of commencing the action plan drafting. So I think that's the first thing I usually mention during the call. I think the main purpose is to try and find out if there any sort of potential impediments to the action plan proving successful, if there are any sort of barriers in the workplace, sort of difficult or challenging professional relationships that are sort of impeding the [doctor]. So that's one of the reasons for the call, and that's why it's important that the [doctor] does feel comfortable and knows that we're not going to sort of repeat that they've said […] without their express consent. (Professional Support Staff)

		Yeah, I think I must have approached in like ST1 or 2, and it was a time when I was still trying to find my feet in my training programme and I didn't feel very comfortable yet, and I was trying my best to give a good impression at work and give off the air that I felt like I was settling in and coping well, when I didn't really feel that way all the time. So having concerns about how information is shared is quite a big deal I think and worrying that suddenly your educational supervisor's going to be sent a report for updates about anything is a nerve-racking. But I don't think I had those worries for very long, I think that they do clarify the process quite early on and I was never aware that anyone clinically who I worked with had any idea that I went to the PSU, except for when I ended up mentioning it, so that was quite good. (Doctor)<br><br>They worry about references and how they're seen by others is more important, they're seen in the short term as competent, for me I'm in post, if someone sees me as a bit rubbish for a couple of years fine, it's not the end of the world is it. (Doctor)
Section 3: Self and situational awareness		
15	3.2	***When the doctor is able to explore an issue from different perspectives with a trusted facilitator (C) a process of reflective reasoning (M) leads to greater situational awareness and perspective (O).***
		So I think it does, and the outlook as well changes, the outlook of I'm an anomaly who's not fitting into the training system, let's go and sort this out and step back into it, actually there has been a change in mindset, this is a training system which is imperfect and I've had to work out the best way of making it work for me, of fitting in without making myself fit in. (Doctor)<br><br>So often lots of short open questions, and I think perspective questions, really thinking about who else is involved, what's their position, what do they notice when this is happening, what ideas do you think they might have about this, if they were in the room listening to our conversation right now what might your supervisor say, who have we not talked about that might be relevant, what about your wife, if she was hear what would she say she's worried about, what else has she noticed about how things have been. Constantly inviting them to take a different position around the central problem that they've come with, they often come with a very fixed problem they can't think outside of, and what we do in those early meetings is layer lots of questions around the problem, so it's rarely about what are you doing, what's the problem, through I think use of language, non-problem related language, try and avoid that, language that opens up perspectives and possibilities, and a stance that says, if someone's used to people go down a rabbit hole they get very hooked on one idea and they get very seduced by one issue, and just keeping

		that very light touch. So the jargon I hold is jargonistic, but holding multiple hypotheses at any one time, not just one, someone comes to me with a problem, there are multiple hypotheses, and we just have to enquire into all of them, and then you need to figure out between you and me what's a useful way forward, not what's the right way forward, what would be a useful next step, that's all I'm thinking about. (Professional Support Staff)<br><br>I think it's that objectivity, and often I'm quite self-critical and think there's a reason this happened and it was because of me and how I am, and I think it gave me that sense of actually it's not necessarily what I've done or how I am but it just gave me that objectivity that I didn't have before. (Doctor)<br><br>I don't think it shifted my understanding but it allowed me some form of acceptance of what was happening, and also allowed me to get a better perspective on the all the things, all the stresses that I was facing that were effecting my ability to work. (Doctor)
Section 4: Motivation and engagement		
29	4.4	***When the doctor's employing organisation takes responsibility for ensuring the process and actions are swift (C), timely (C) and clearly communicated to the doctor (C) the doctor is more likely to feel that resolution is achievable (M) leading to a greater motivation to continue to engage (O).***
		It took a good few months to get it signed off, and I think it was sent to us December 2021, and I have no idea whether it was delays in getting people to come to the meeting but it took longer to discuss and sign it off, and finally signed off in April 2022. I don't remember what the delay was due to, but it was such a protracted process, but it was not because of PPA, it was our trust which was dragging its feet for whatever reasons. The clinical lead and director within pathology were not proactive in doing things that were required of them, for example, if they had found out right after the PPA plan came in; so there was this disconnect between the trust and my own clinical lead and director, they kept saying we have not been involved in implementation of this plan, it's the trust's directorate, and the associate medical director she has discussed it with my clinical director and that she's in agreement or whatever, and they will be looking for places for you to go. So there was this thing going on and a month goes by and you're still thinking ok so why haven't we found a place, so we come to the next meeting and we haven't got anything to say. So it went on for a time and finally when they did make phones calls nobody was sort of accepting, it's difficult, I understand why they were saying no, but the delay I think was from my own people not making the sort of contacts. (Doctor)

		Because for almost a year I felt that there was no way out of this situation, it was carrying on for such a long time, so finally when PPA called me and said this is how it is going to be, and at the end of the plan we will review the situation, and the trust will be fed back as to what happens. And so the first thing was that there was going to be a plan, and that there was going to be a defined end point, it may not be immediate but at least there was one, which made me feel that there was finally light at the end of the tunnel. (Doctor)<br><br>But again we don't have full sight of it, and I don't have to tell you, often less than suboptimal conditions in the relationship between the parties and residual things and the working environment if there's no placement for whatever reason in the plan. So in some ways you sometimes you might think the plan that we've constructed here is really, really good but we're now handing it over into a less than futile environment, and you think if only a few things were slightly different I would feel a lot better about this, but for us it's kind of handing things away to a certain extent. (Professional Support Staff)
Section 5: Personal and professional growth		
42	5.3	***When the facilitator exposes the doctor to similar examples of where professional support has been required (C) and / or made to understand that these experiences are common (C) a process of normalisation (M) results in greater perspective (O) and less shame (O).***
		I would like in that situation for them to come out of the room feeling like I know where to go, I know how to speak up, if I'm struggling, I know where to get help. Because it's not just about that particular episode, it's an opportunity then to affect the way that they practice going forward, to be able to nip things in the bud, but also if things do happen then to be open and honest about it and seek help, and CPD if that's what's required, or support or whatever. (Professional Support Staff)<br><br>Yeah, and also I think he was experienced and qualified in a way that a lot of other people aren't, cos some people can just be doctors or therapists, but he was also a coach and I think he brought a lot of his coaching knowledge into our conversations without me knowing at the time, which helped me think things through in a different angle, and feel more empowered with my decisions and moving forwards. (Doctor)<br><br>People will say 'oh I thought I was the only one', or 'that's so comforting to hear that other people are going through the same thing.' ...I will say …'normally what happens in these types of situations', so I do find myself saying that a lot…'I'll also say obviously we set up this service because there was such a huge demand, and unfortunately there was a

		big demand, but you'll know that you're certainly not the only one going through some issues.' (Professional Support Staff)<br><br>It was probably just by reflection, with help from the counselling sessions, I think the counsellor at the end says I don't really need to tell you much cos you know what you've got to do, and that's how we're going to end it. So I suppose the counselling gave me more of a confidence and empowerment to do it, while I probably knew going into it what I wanted to do, but the counselling empowered me to do it. (Doctor)<br><br>And then when it came to a point where I had to speak to my educational supervisor about it, the lady at PSU helped me, talked to me about that, cos that was a huge thing that I didn't want to do and was very worried about, cos to me it's a boss, I know they're meant to be pastoral. (Doctor)
Section 6: Culture of organisation		
45	6.1	***When the doctor perceives that taking time off will be judged as being 'lazy' (C), they may feel guilty (M) and fear the associated stigma (M) and delay seeking support (O)***
		I felt really guilty having sick leave, and obviously if I just spoke to people in my family, or friends and other colleagues, they tend to have a similar opinion of you know you're being lazy, are you sure it's that bad, all this stuff, especially when it's mental health related. (Doctor)<br><br>And I think because there was actually somebody off at the same time with, to subsequently not return to work and resigned, I think that culture meant a little bit that I was being weak and lazy, and I think that was something that I had overheard from a colleague, or a colleague had overheard in a coffee room, and that was pretty hard to deal with but I think it feeds into that culture. I hope it's changing now, it feels like, and I think I would still continue to have really good support if I was to have the time again for example. (Doctor)<br><br>So I just felt one day I can't do this, I don't want to do this, and took myself off to my GP and was signed off, and that felt like a big unburdening but felt a very big step, felt a very negative step as well, particularly worrying about letting your colleagues down, cos you know it's such a shit show that one of you goes off and then the pressure gets ratched by another 12.5% because one of the eight has disappeared. (Doctor)
