# Supplementary material for: Optimising professional support for doctors who experience work performance issues: a realist evaluation
Source: BMJ Qual Saf. 2026 Apr 20;35(8):e019749. doi: 10.1136/bmjqs-2025-019749 (PMC13479272; doi:10.1136/bmjqs-2025-019749)
Supplement: Supplementary file 5 [file bmjqs-35-8-s005.pdf]

UNIVERSITY OF PLYMOUTH

A Guide to Optimise the Delivery of Professional Support in the NHS

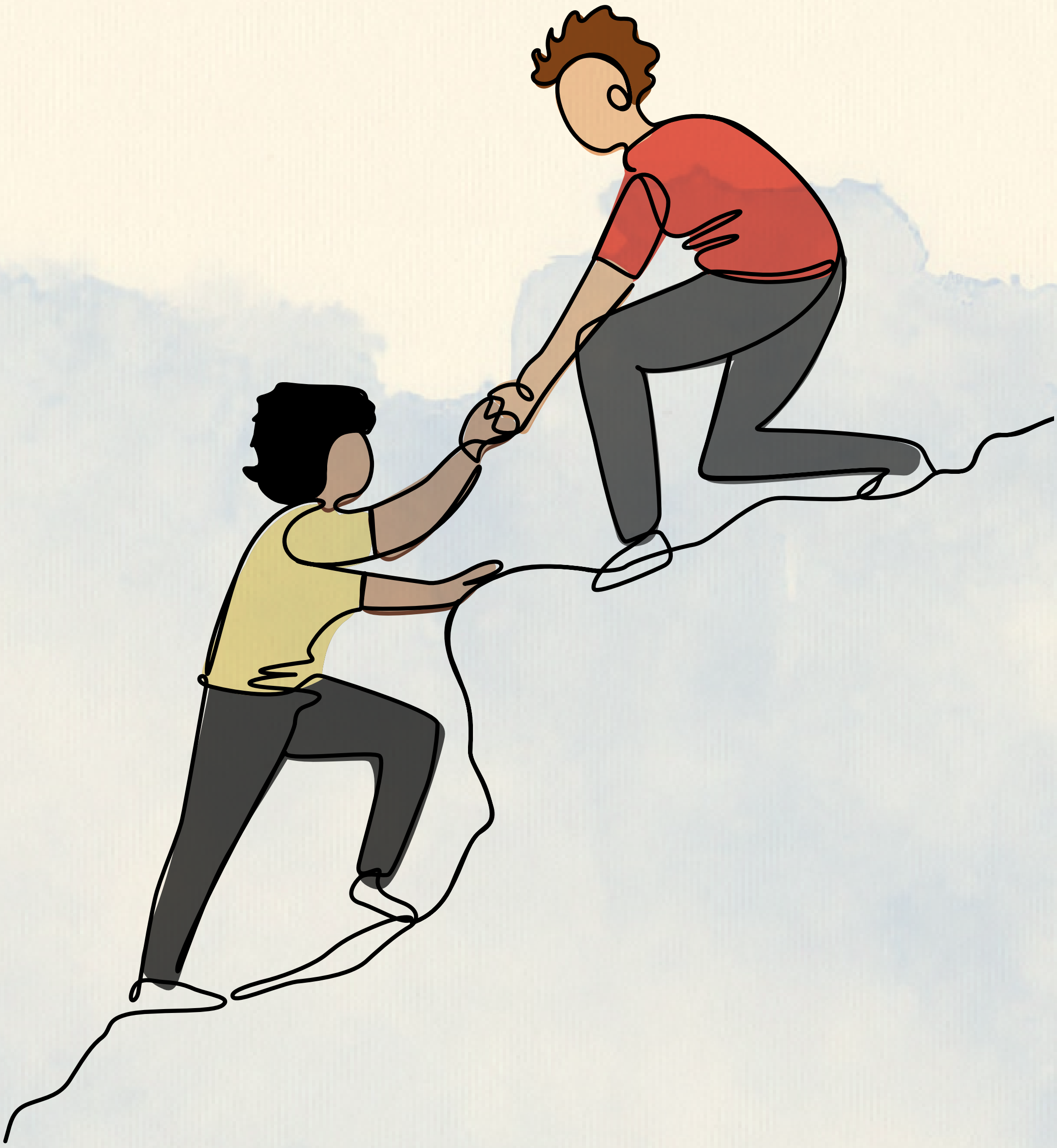

Contents

About this guide 3

What is this document? 3
Who is it for? 3
How did we develop it? 4
Terminology 4

The professional support pathway 5

Summary of key principles to optimise the delivery of professional support services 6

How to make a difference 7

1. Pre-support phase 7
2. Support phase 17
3. Post-support phase 31

Find out more 34

Linked publications 34
Acknowledgements 34
Funder statement 34
How to reference this document 34
Sources of support 35
Links 36

About this guide

What is this document?

This guide is centred on the experience of doctors in professional support. It tells service providers, whether within Trusts or external, what doctors have told us about what does and doesn't work for them, when it comes to professional support.

The guide follows the 'pathway' of professional support, spanning from the initial point of a doctor realising they may need help to the point where the doctor can work without their performance being affected. We have identified three stages in this pathway: pre-support, support and post-support or reintegration. We also highlight issues that span the entire length of the pathway.

Who is it for?

This guide is designed to help departments, Trusts, services and individuals that lead and deliver professional support services to understand the key actions you can take to optimise the delivery of professional support services for doctors. The document aims to help you reflect on your current service and identify areas of improvement, as well as recognise what you are doing well. It also offers practical steps that can be taken should challenges arise.

About this guide

How did we develop it?

The guide forms part of an NIHR-funded research project that ran for 4 years, called RESTORE 2, completing in June 2025. As part of this project, we interviewed doctors who received any type of support, whether that was for help returning to work following leave, mental or physical health issues, or in response to concerns about performance or behaviour. We also interviewed those providing professional support within Professional Support Units, NHS Trusts and a professional support organisation about the work that they do. The recommendations are based on best practice in those sites. We used a realist approach, which asks what works, why and for whom (rather than simply whether it works). This has resulted in an evidence-based, nuanced understanding of the problem and the identification of solutions to optimise professional support in the NHS. It is the first guide of its kind in the field.

Terminology

For the purposes of this project, we conceptualise professional support very broadly on a spectrum from the less serious issues that affect a doctor's health and wellbeing, e.g. requiring input on exam support, to the more severe end of the spectrum where a doctor may have serious performance or behavioural issues leading to patient safety concerns. Where there is a severe performance or behavioural issue, this is also commonly called 'remediation' or 'performance management'. We do not use these terms because of the negative connotations associated with them which perpetuate stigma, potentially preventing doctors from seeking help. The term 'professional support' reduces this stigma and also emphasises the responsibility of the system rather than just the individual in the process.

We use the term 'medical education team' to refer to anyone who provides professional support to doctors based in Trusts, either in training programme's or otherwise. This could include those who carry out appraisals, Clinical or Educational Supervisors, Responsible Officers, Directors of Medicine, Medical HR roles, etc. A resident doctor is a fully qualified medical professional who is either currently in postgraduate training or gaining experience as a locally employed doctor, aiming to become a consultant, GP, or specialist.

The professional support pathway

This guide is a resource for those leading and delivering professional support in Professional Support Units, Professional Support and Wellbeing Services, NHS Trusts and other professional support organisations. As such, its aim is to encourage reflection on the service you already provide, from the pre-support to the post-support phase, to enable you to identify areas that are following good practice and those that could be improved.

In each phase, we outline a number of key principles presented from the doctor's perspective, along with recommendations on practical steps to address these principles. It is important to note that the support phase should only be carried out by those trained and experienced in the techniques described.

During the research, several overarching areas were identified as being of crucial importance to the effectiveness of professional support. We urge those providing professional support to ensure that these principles are prioritised.

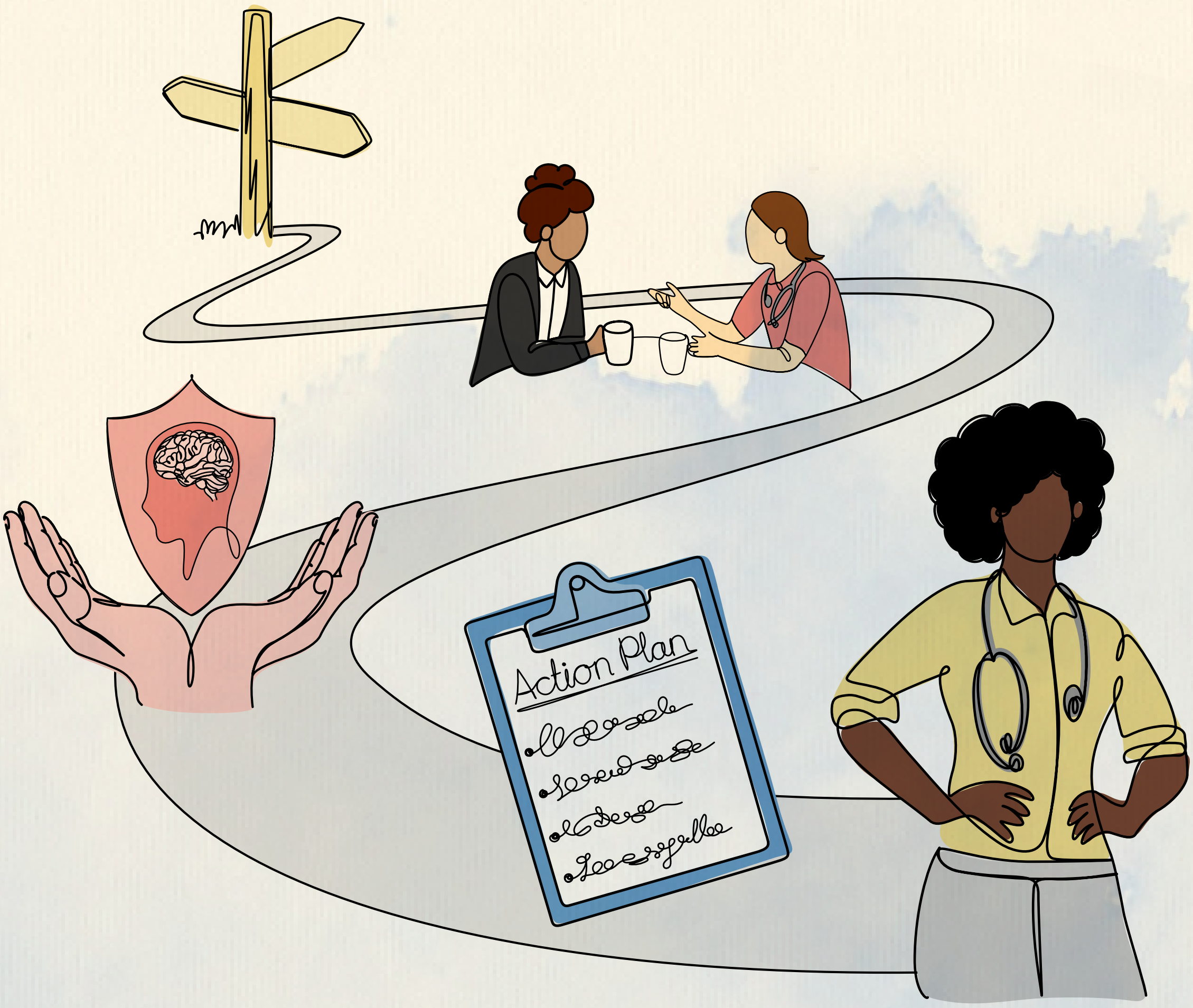

Summary of key principles to optimise the delivery of professional support services

1. Early identification and provision of support to doctors who are struggling can prevent more serious issues requiring performance management later on (section 1, pre-support phase).

2. Psychological (emotional) support is essential throughout the whole professional support pathway and cannot be considered an optional extra (section 2, support phase).

3. All staff supporting doctors need to be aware of the whole professional support pathway (sections 1, 2 and 3) and not just the parts of the pathway that are relevant to their specific role. All staff includes (but is not limited to) educational supervisors, Medical HR personnel, Occupational Health, Responsible Officers and Professional Support Units/Professional Support and Wellbeing Services.

4. In Trusts, a named person should have the responsibility and oversight of the entire professional support pathway for doctors. Consider developing a Non-Executive Director (NED) role, responsible for making professional support visible within the Trust.

5. There needs to be equal access to support for doctors in formal training programmes and those who are not. Support needs to be available and easily accessible to locally employed doctors as well as autonomous practitioners such as hospital consultants, Specialty and Specialist (SAS) doctors and GPs.

6. International Medical Graduates (IMGs) are a particularly vulnerable group, and are being referred for professional support and performance management at a disproportionate rate. All organisations should ensure IMGs are well-supported, and services must proactively and pre-emptively address common concerns that IMGs may have.

How to make a difference

1. Pre-support phase

1.1 Many doctors we spoke to didn't know what support services may be available to them when they first realised they needed support. Whilst there are many services available for doctors, it is often unclear where they can find this information or how to decide on the most appropriate service for their needs. A doctor we interviewed told us:

"Laying out what the support is clearly and concisely so people know… there are so many different agencies, routes and ways into it that it's such a mess at the moment." (Resident Doctor)

Recommendations:

- Advertise services as widely as possible to doctors and those who may be able to signpost doctors to your service (e.g. through educational supervisors, clinical directors, training managers, etc.). NHS Trust inductions are a good opportunity for this, especially for IMGs who may not be familiar with the type of support available.
- Let doctors know that the service is free.
- Ensure communication about the service is targeted and inclusive of different types of doctor, such as IMGs or doctors with neurodivergence.
- Annual appraisal documentation should include signposting to professional support services.
- Keep your staff intranet up to date with working links to sources of support.
- Perceptions matter too. Think about how information about the service may make the doctor feel – is it a 'naughty step' for someone struggling, or an opportunity for them to learn the skills they need to flourish in their career?
- There may be a number of misperceptions about professional support, particularly amongst groups such as IMGs. It is important for these misperceptions to be pro-actively addressed by services.

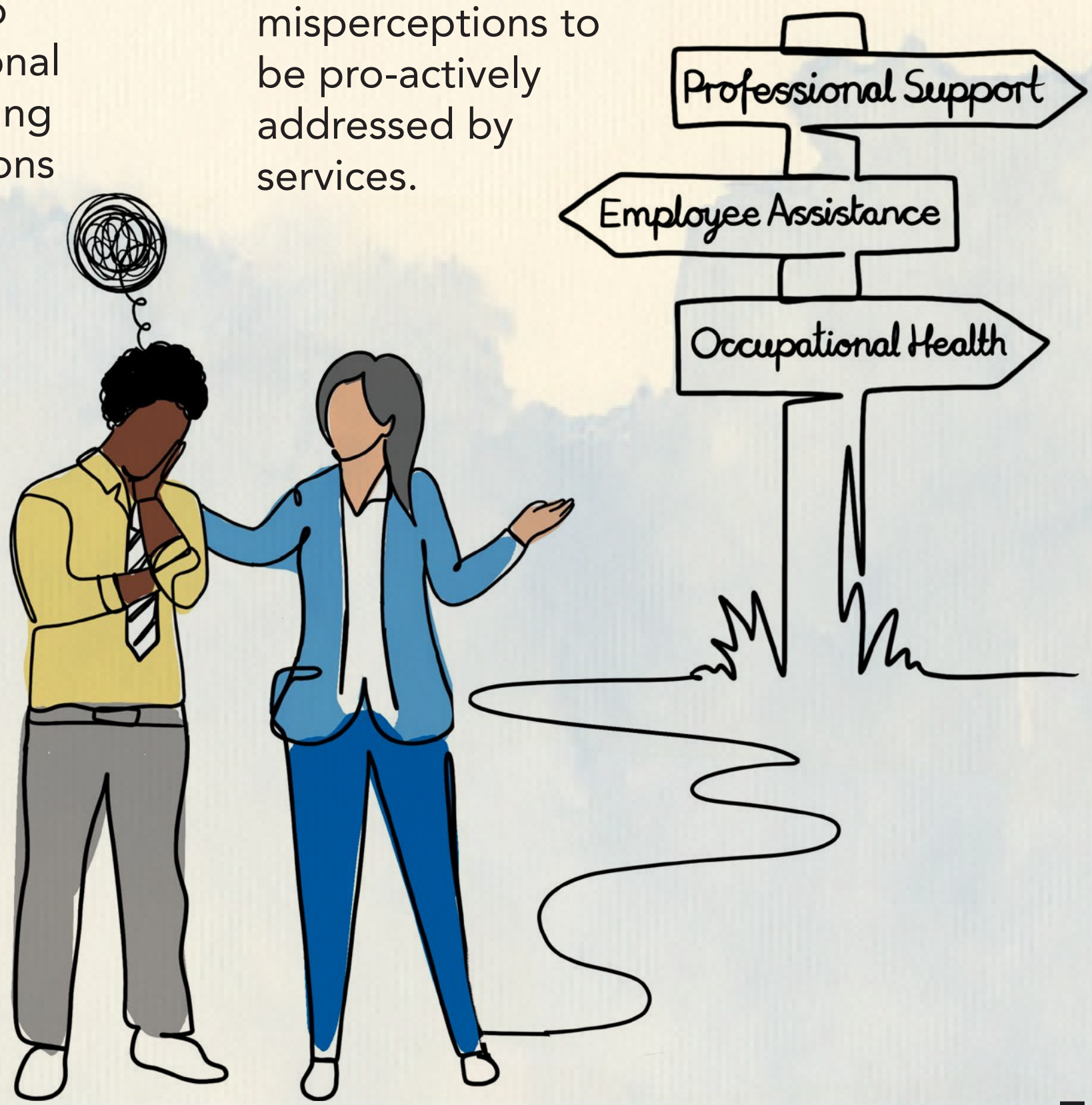

1.2 Doctors have told us it was important for them to be able to self-refer to services, which allowed them to stay in control of the situation and their experience.

"I referred myself. I think that's the right thing to do and it's a stronger thing to do; it gives one a bit more control as well over a situation." (Consultant)

Recommendations:

- If your role involves supporting doctors, ensure that you know how to signpost them to access support and can easily do so.

- Normalise accessing support. It can be helpful if senior doctors' role-model their experience of accessing support (by speaking about it publicly, for example).

- Recognise the importance of doctors being in control of the situation. This empowers them to be in charge of their experience and encourages engagement in the process.

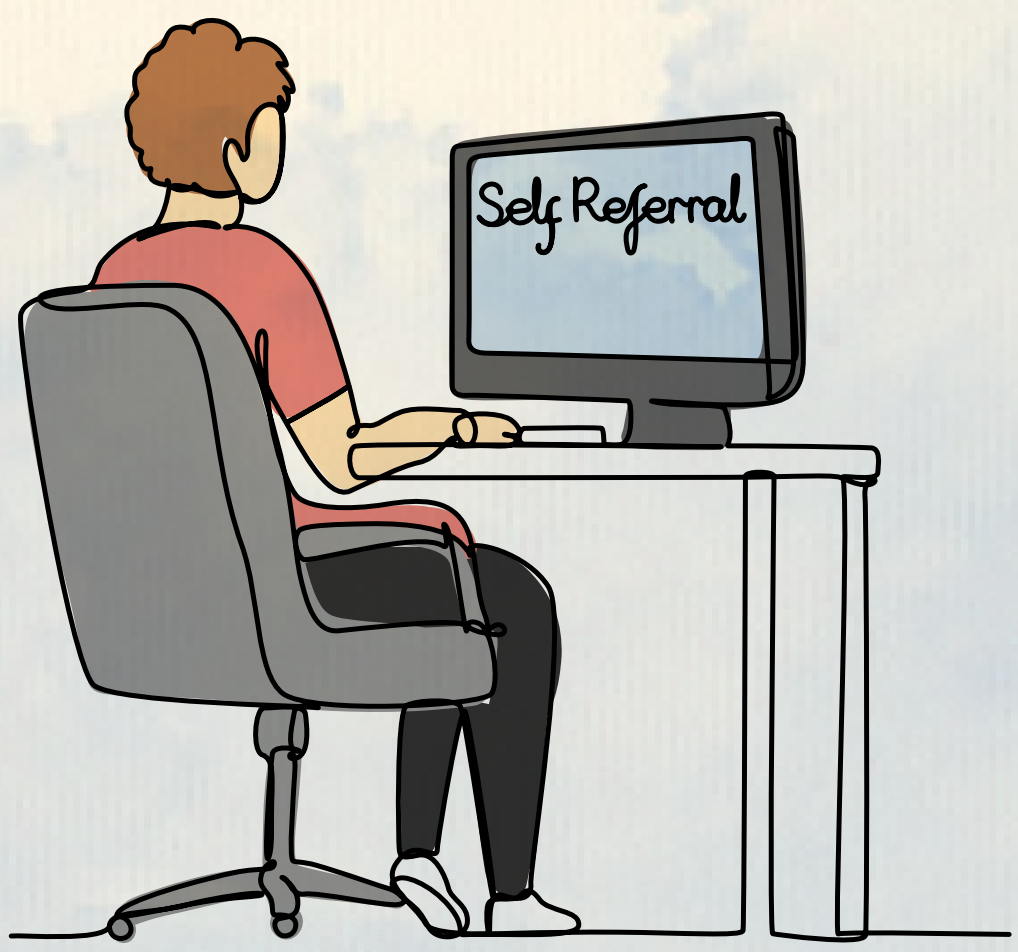

1.3 Doctors experiencing burnout, depression or an undiagnosed neurodivergent condition may find it difficult to self-refer to services. Doctors have told us that when they've been in these situations, they appreciated a person they trust making a referral on their behalf.

"I think that having somebody tell me what to do, recognising that you were vulnerable, weak and sort of drifting, was quite a relief in some ways because it felt like I wasn't making the decision to have time [off], it was someone telling me I needed to take time off." (Consultant)

Recommendations:

- Consider the possibility that a doctor may be experiencing difficulties and uncertainty in their personal or professional lives, or they may be too unwell to recognise their need for support.

- Be aware of what resources may be available in addition to Occupational Health for doctors who are unwell. Doctors have told us that Occupational Health services are variable, and they often prefer to access external services such as Professional Support Units or the NHS Practitioner Health Service as they are independent of their employer and are focused on the needs of doctors.

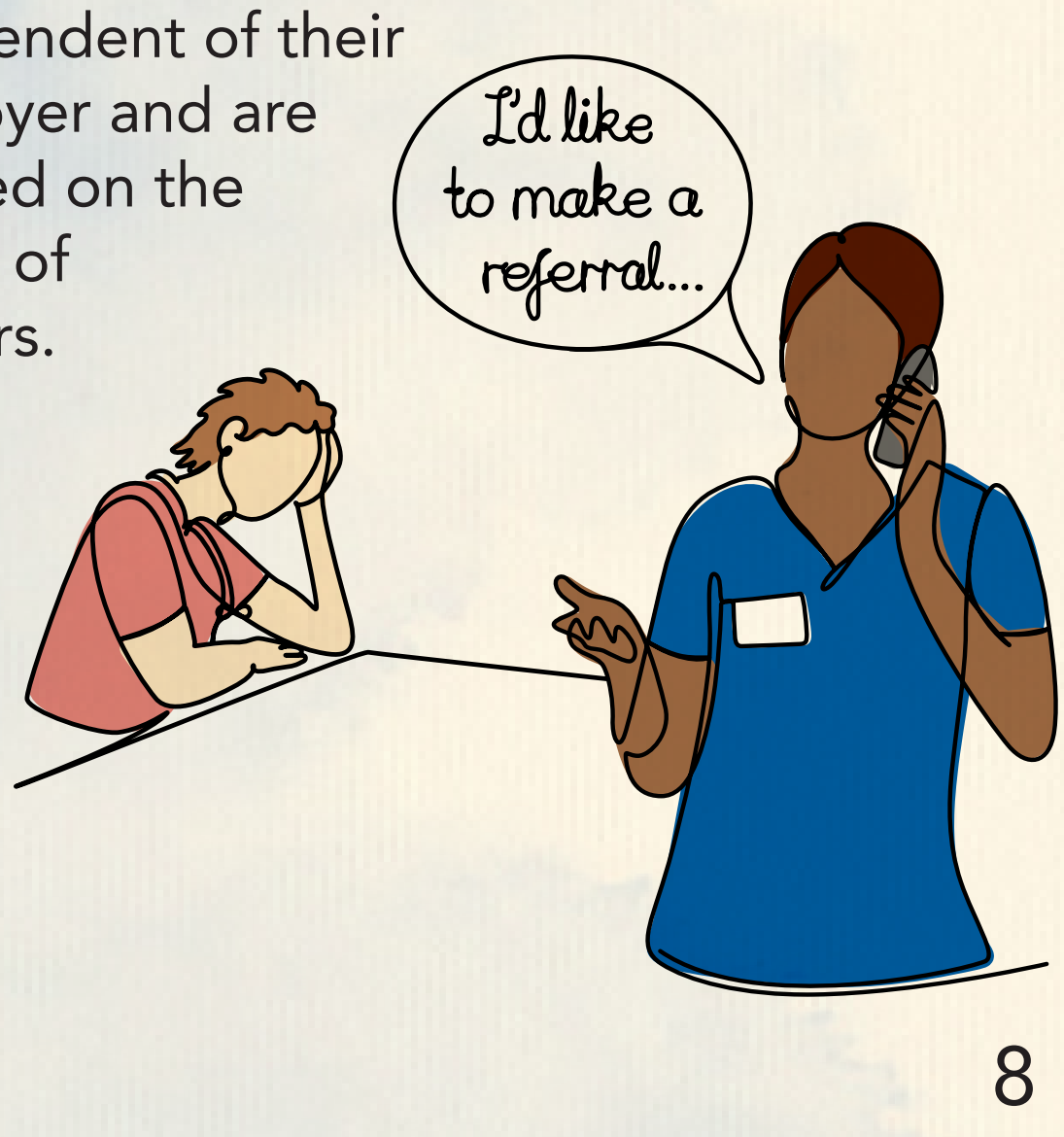

1.4 Doctors have told us they appreciate a short waiting time for support. The possibility of a long wait for access to services may put doctors off from contacting them.

"It was really good to have access to that without having to wait and without having to pay." (Resident Doctor)

Recommendations:

- Ensure prompt responses are a priority in any service.
- It may be necessary to invest in sufficient administrative support to provide timely responses.
- It may be helpful to communicate typical waiting times for access to different services.
- If it is likely that a doctor will need to be referred to other services (e.g. Maintaining High Professional Standards (MHPS) or Practitioner Performance Advice), it is best to refer them earlier rather than later, so required support is not significantly delayed. This also prevents the doctor from becoming deskilled.

1.5 Doctors appreciate an outline of what they may expect from a process or service. This encourages openness and permission to explore the doctor's needs and expectations, as well as helping to reduce uncertainty.

"I think when I accessed [the service] when I was feeling really unwell, it was very clear about what would happen and the timeframes... and waiting lists, and things like that, it was all very clear cut. I think that is really helpful, necessary even, because I think again if you access something like that you expect everything to take months, and it wasn't months, and I knew that if it was longer than a couple of weeks that I'd heard from anybody then that would be an issue that I could raise." (Resident Doctor)

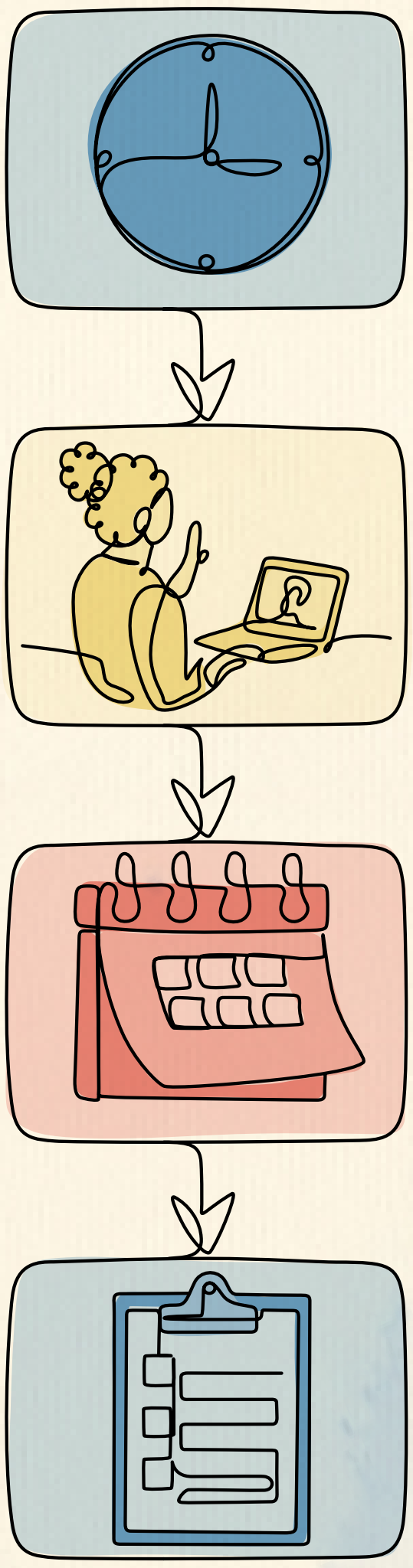

Recommendations:

- Tell doctors when they initially access the service what they can expect and what is not provided (e.g. legal support).
- Doctors may find it useful to be provided with a flowchart, outlining what is going to happen and any steps they need to take.
- Provide this in written form inclusive of the doctor's specific requirements, so they can refer back to it if necessary.
- Ensure there is enough information in any confirmatory texts or emails sent to the doctor.
- Ensure the doctor is provided with a contact email in case of enquiries.
- Timings are important. Give the doctor a realistic timeframe about when key activities are going to take place.

1.6 Whilst some doctors told us that they trust the service to protect confidentiality, this is often a big concern for other doctors, particularly if they are resident doctors in a training programme.

"I must have approached [the professional support service] in ST1 or 2, and it was a time when I was still trying to find my feet in my training programme and... give off the air that I felt like I was settling in and coping well, when I didn't really feel that way all the time. So having concerns about how information is shared is quite a big deal, I think, and worrying that suddenly your educational supervisor's going to be sent a report for updates about anything is nerve-racking. But I don't think I had those worries for very long; I think that they do clarify the process quite early on." (Resident Doctor)

Recommendations:

- Independence of a professional support service from their place of employment is important to doctors who may feel concerned about confidentiality.
- Doctors who access services provided by Professional Support Units may not realise that they operate independently of NHS England/their training programme and may need this explained to them.
- Confidentiality is often a big concern for IMG doctors. It is important to address some of the misconceptions around accessing support that these doctors may hold.
- Regularly reiterate to doctors that anything they tell a coach or facilitator will be kept confidential unless there are exceptional circumstances, such as a concern about patient safety.
- Remind doctors that anything that they put in writing (email, Teams messages, WhatsApp on work phones), is disclosable. It is best to store communication in a non-work email account.

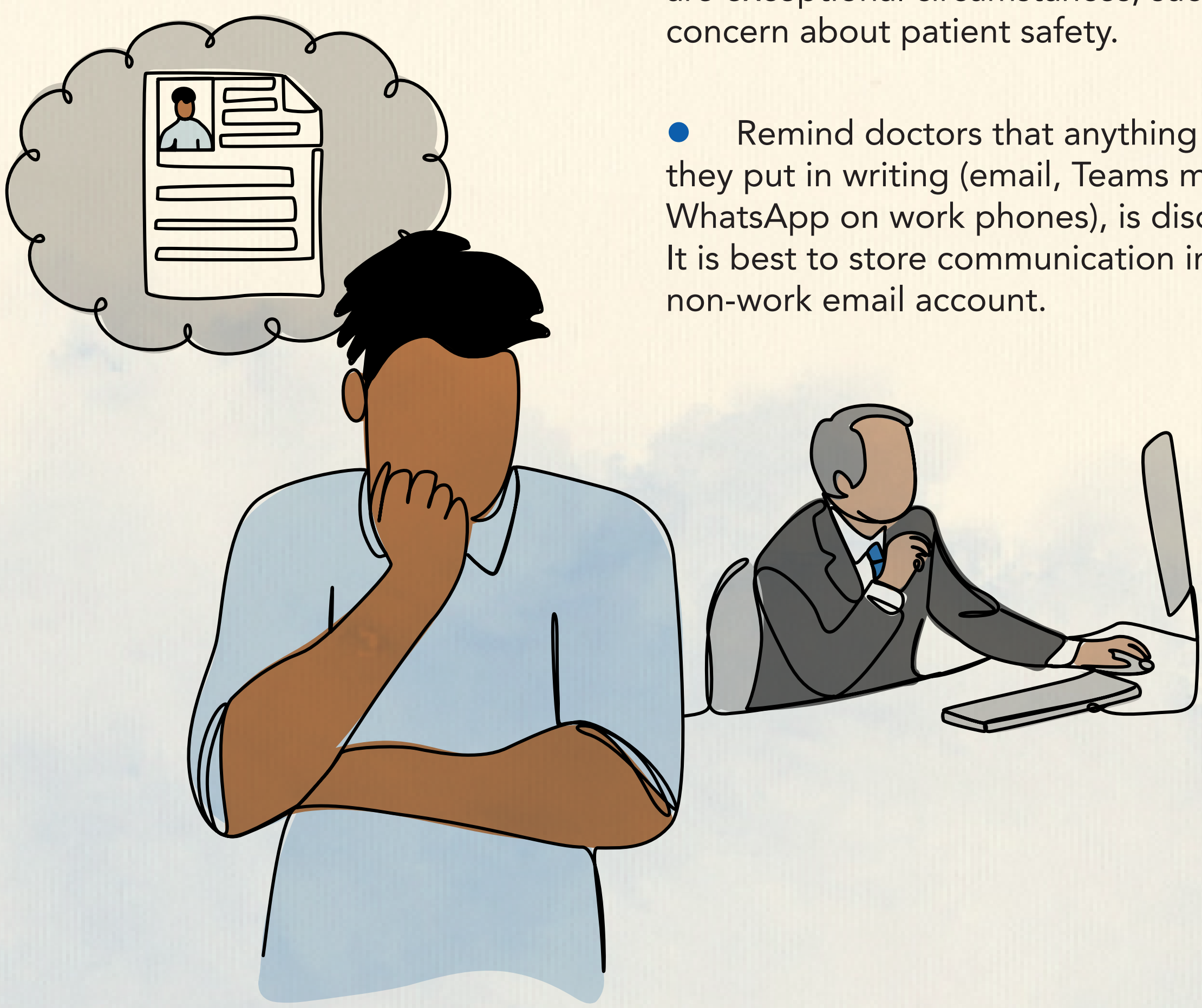

1.7 How professional support is framed is an important part of how doctors experience it and their role within it.

"I think it was definitely advertised as a positive experience, and the people that I spoke to, all spoke about it as a positive experience and a way of bettering yourself. It wasn't if you're struggling you should do coaching, or if you're having a hard time at work, it was more it just made me a better clinician, and loads of people are doing it, it's really common." (Resident Doctor)

Recommendations:

- Professional support can be viewed as an opportunity for doctors to develop awareness of their strengths and develop confidence, allowing them to move forward in their career.

- If a doctor doesn't agree with the need for their referral, encourage them to consider professional support as an opportunity to demonstrate and enhance their skills.

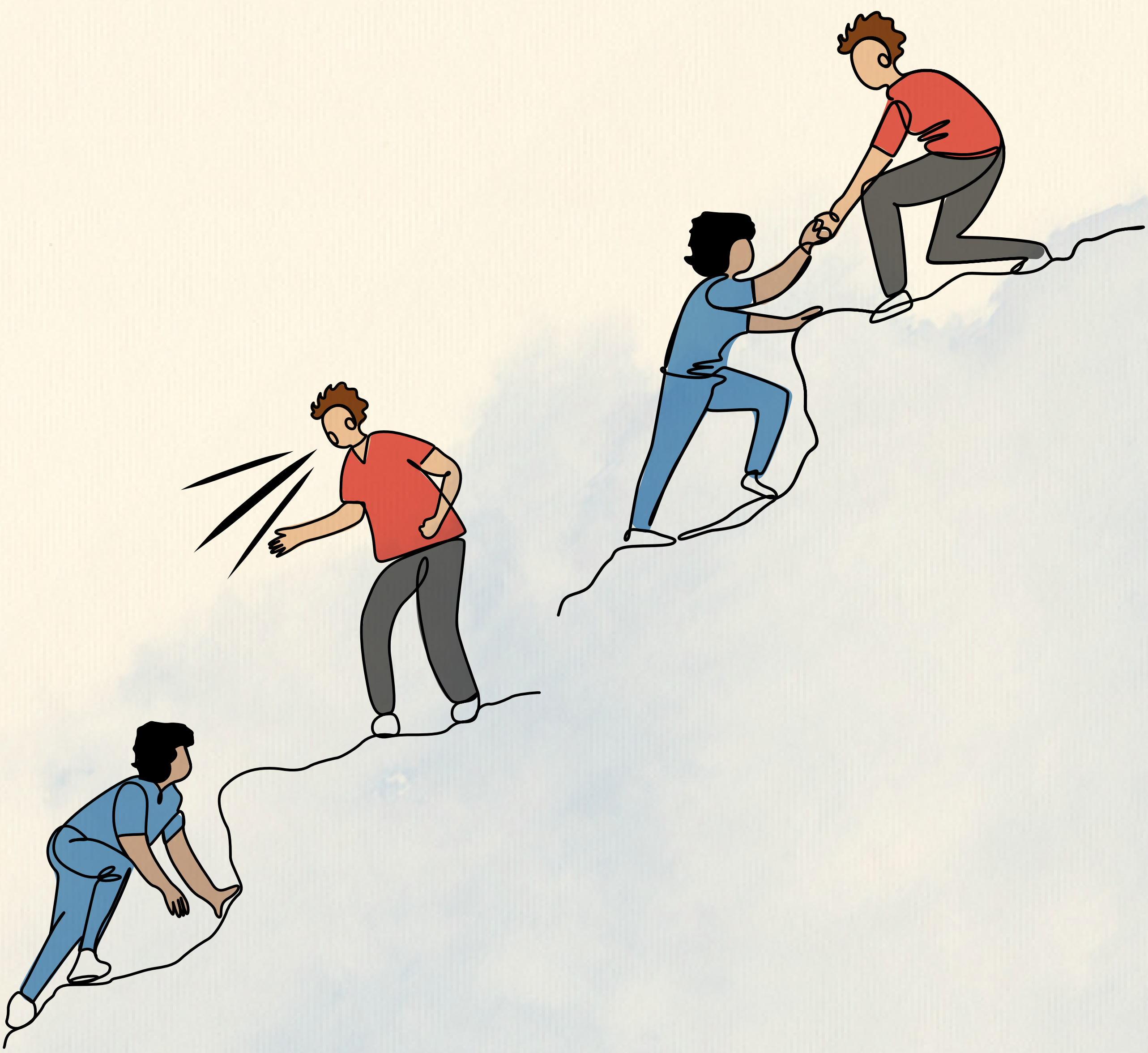

1.8 A doctor's professional identity can have a big impact on how they engage with professional support. Doctors may feel a sense of shame when they realise they need support. This can lead to a delay in accessing support.

"I'm sure lots of doctors felt the same in that I felt quite embarrassed and ashamed that I was having a difficult time and wasn't really holding [it] together." (Resident Doctor)

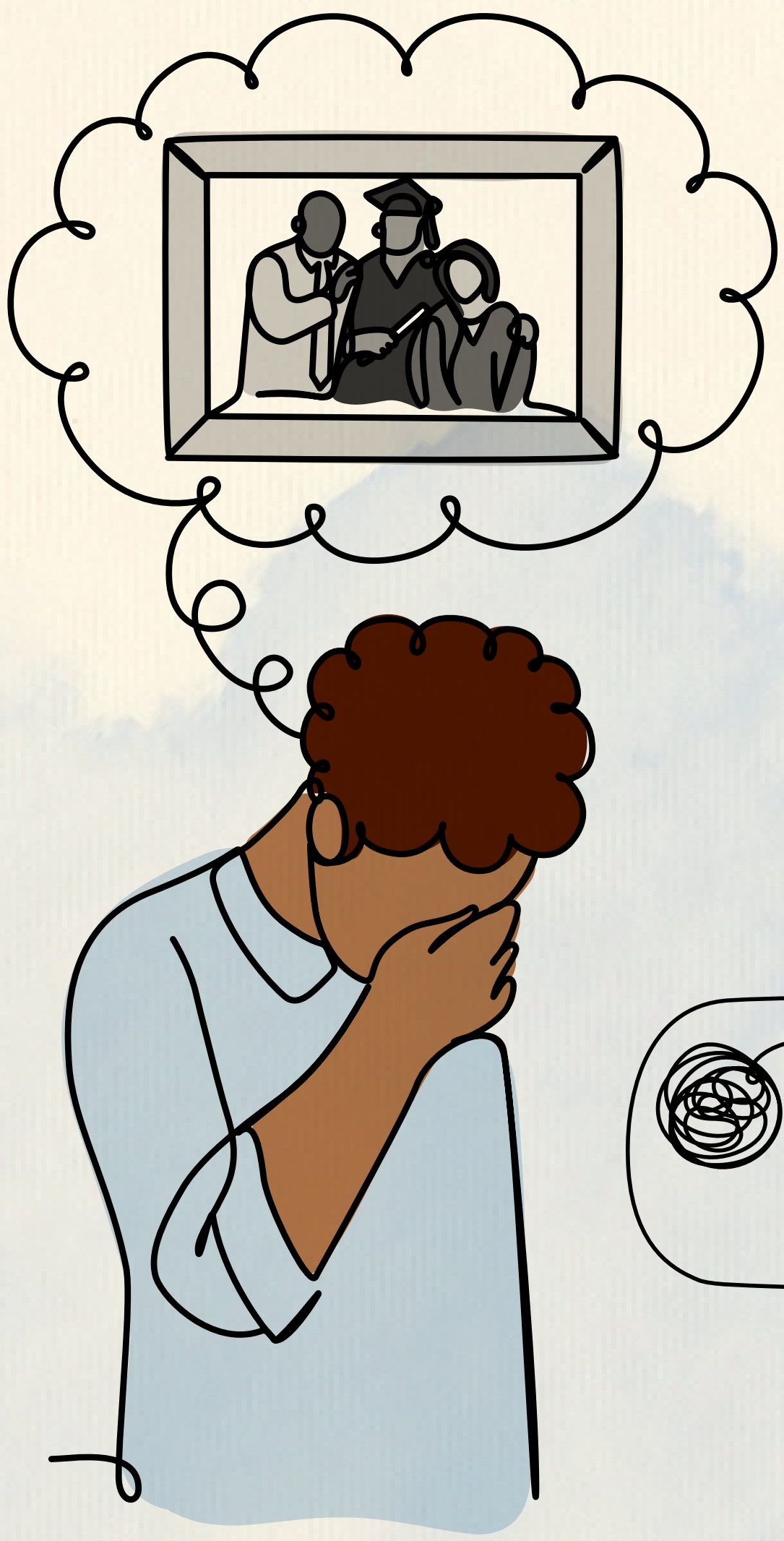

Recommendations:

- Normalising the experience can help doctors feel like they are not alone in their need to access support. Telling a doctor that many other doctors will need support at some stage during their career can help.
- Hearing the voices of other doctors who have accessed support may help this process. Consider developing an accessible medium, such as online videos, podcasts or workshops, where they can hear peers talking about how they benefitted from accessing support.
- Doctors may feel that they are undeserving of any support. Reassure them that they have every right to access support if they need it.
- Organisational culture and a sense of belonging have a big impact on a doctor's willingness to ask for help when they need it. This can include guilt around taking sick leave or taking time off, fear of letting colleagues down, or the shame or stigma attached to a need for support. Think about messages in your Trust which may compound or challenge these beliefs.

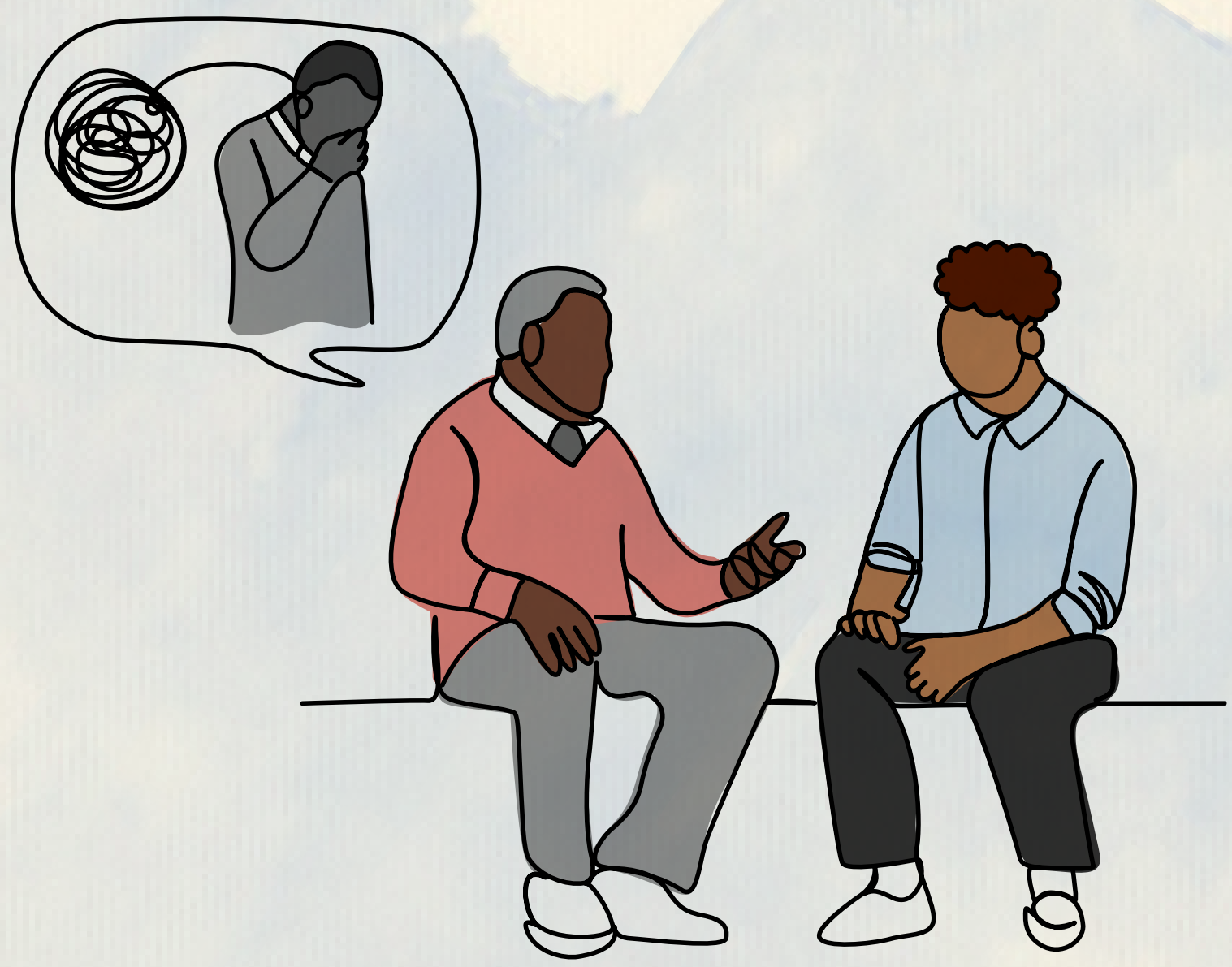

1.9 Going through a personal or professional difficulty leading to a need for professional support can be distressing and isolating for some doctors. It is important to communicate with doctors in a clear and compassionate manner.

“I think that the Trust were very straightforward and approachable, the Associate Medical Director and the HR person, both of them were supportive, very understanding and matter-of-fact, so they never made me feel like the accused person; they treated me fairly.” (Consultant)

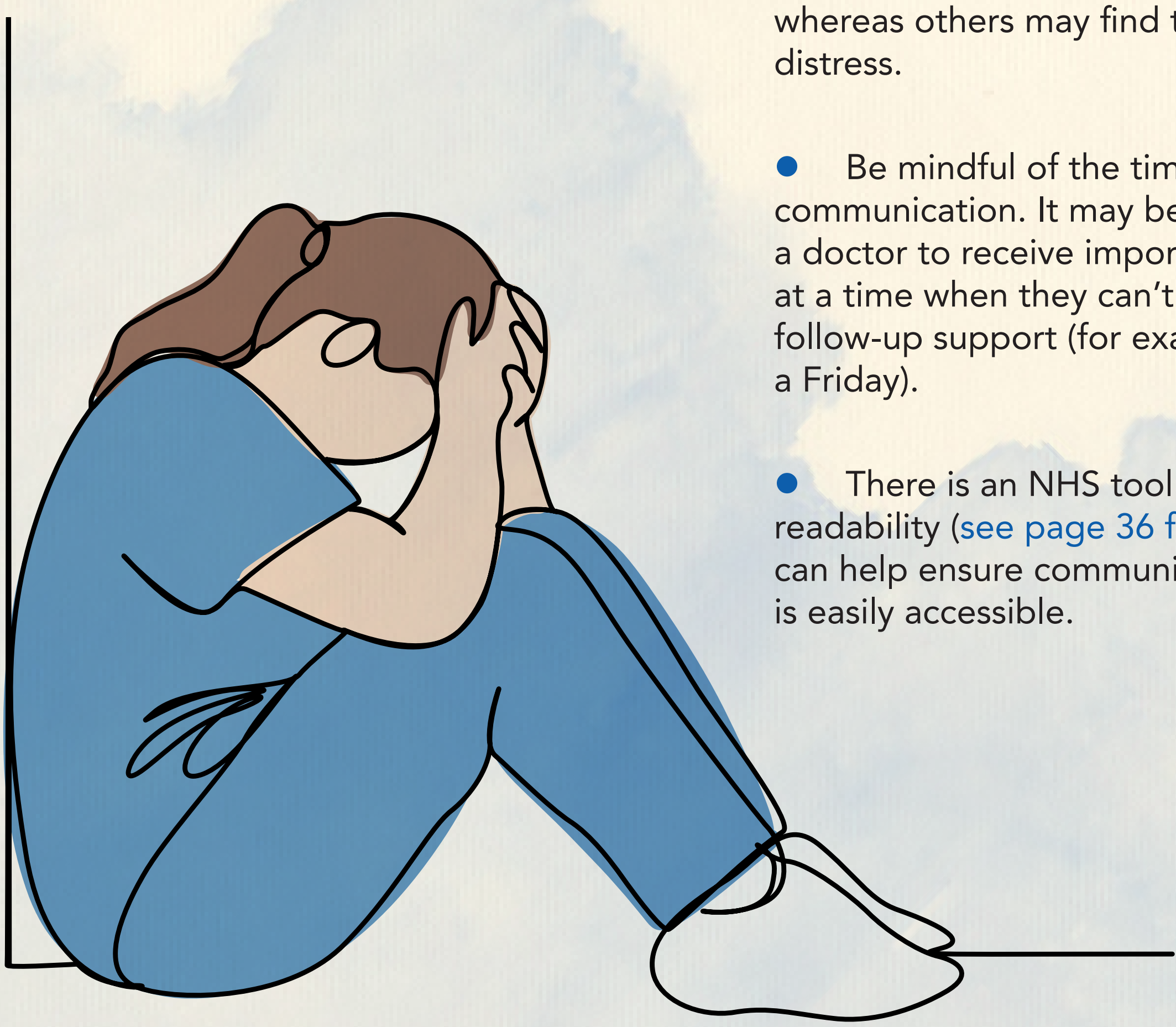

Recommendations:

- Line managers, medical directors or medical HR personnel should co-create a plan of action together with the doctor, including timeframes and the responsibilities of the Trust and the doctor.

- Ensure the doctor is informed of any plans to discuss concerns with external services. Try to keep any written information clear but informal and supportive in tone. Doctors are more likely to stay engaged in the process if they don’t feel pressured or threatened. It can be hard for doctors who are distressed or neurodivergent to make sense of complex written information. Try to ensure there is someone who can verbally reiterate what they need to know.

- Be aware that some doctors may want a lot of detail about what might happen, whereas others may find this increases their distress.

- Be mindful of the timing of communication. It may be distressing for a doctor to receive important information at a time when they can’t easily access follow-up support (for example, at 5pm on a Friday).

- There is an NHS tool to improve readability (see page 36 for a link) which can help ensure communication to doctors is easily accessible.

1.10 Wherever possible, provide opportunities to resolve complaints about a doctor's performance before escalating to performance management processes.

"I think this whole thing could have been avoided if my own clinical lead and clinical director had sat me down. I wouldn't mind if they cross-examined me and said 'what's going on here, we've got this complaint, can you tell me [what happened?]', and it would have been sorted. They were the ones who actually escalated it to the Trust level, which I find extraordinary because I think it's easier to talk to people, to find out; it could be that I had some difficulties and I would have explained everything, but they never gave me the opportunity." (Consultant)

Recommendations:

- Have an informal, non-judgmental conversation with the doctor before deciding on the next steps. It may be that a resolution can easily be found without the situation being escalated.

- Use open questions to encourage the doctor to open up to you.

- In the first instance, the doctor may be more open to talking to someone impartial or external to the situation. This could be a senior colleague from a different specialty or department, or someone external, e.g. from the Professional Support Unit or a Practitioner Performance Advice Adviser.

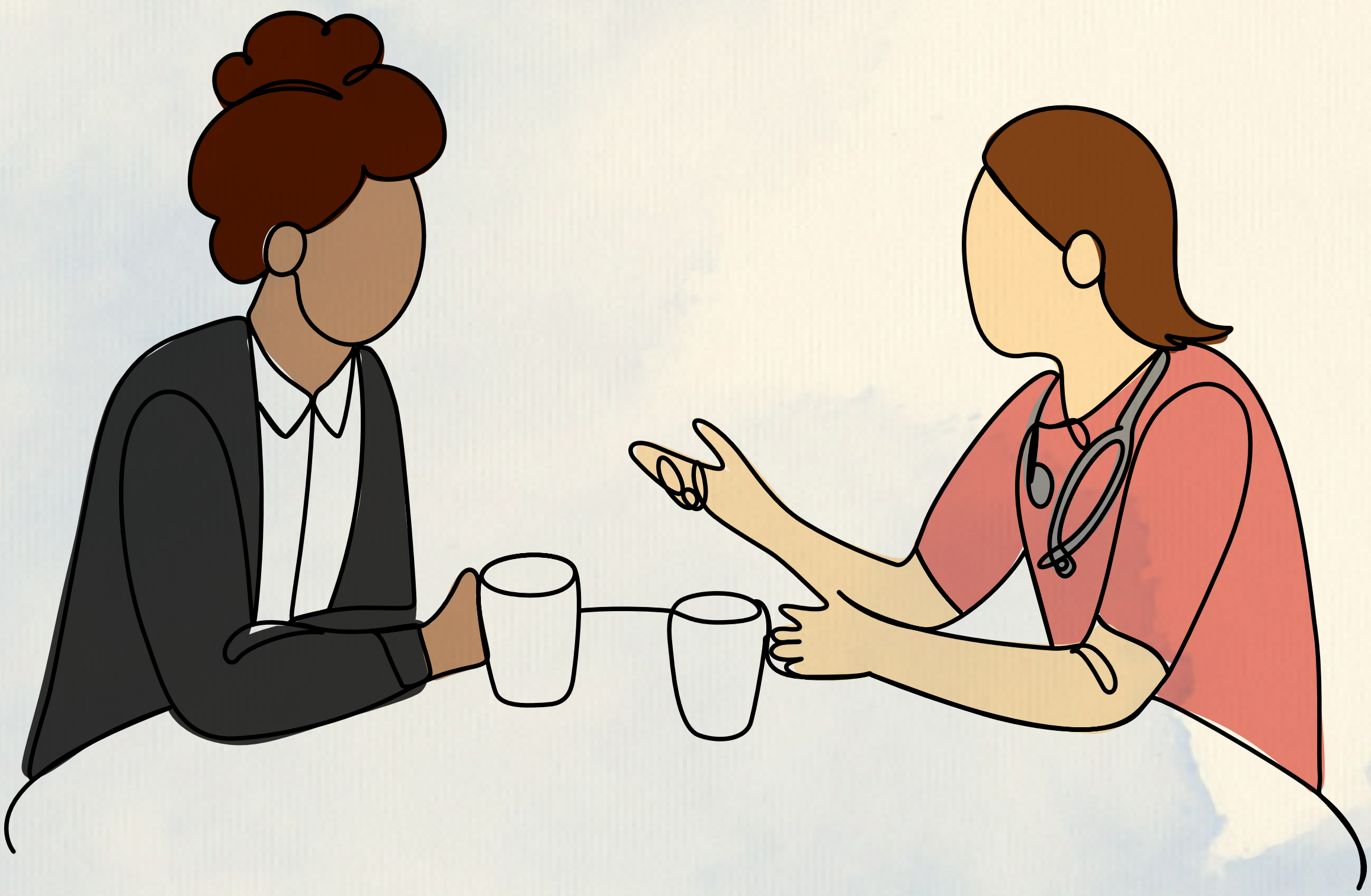

1.11 Provide a support pack for doctors who are involved in formal processes, letting them know what they need to do.

"I was very naive when I went into this process, so I didn't even call the defence union till somebody suggested it to me. You don't think about these things at all." (Consultant)

Recommendations:

- If you are part of a medical education team or in medical management, put together a support pack for doctors who are involved in formal processes, letting them know what they need to do. This is a vital step in ensuring they know how to proceed.

- If the doctor is not a member of a medical defence union, it is still important for them to seek legal advice. They could also contact a Trade Union, such as the British Medical Association (BMA) or the Hospital Consultants and Specialists Association (HSCA), for support.

- Doctors receiving support should have access to a single named point of contact throughout the process.

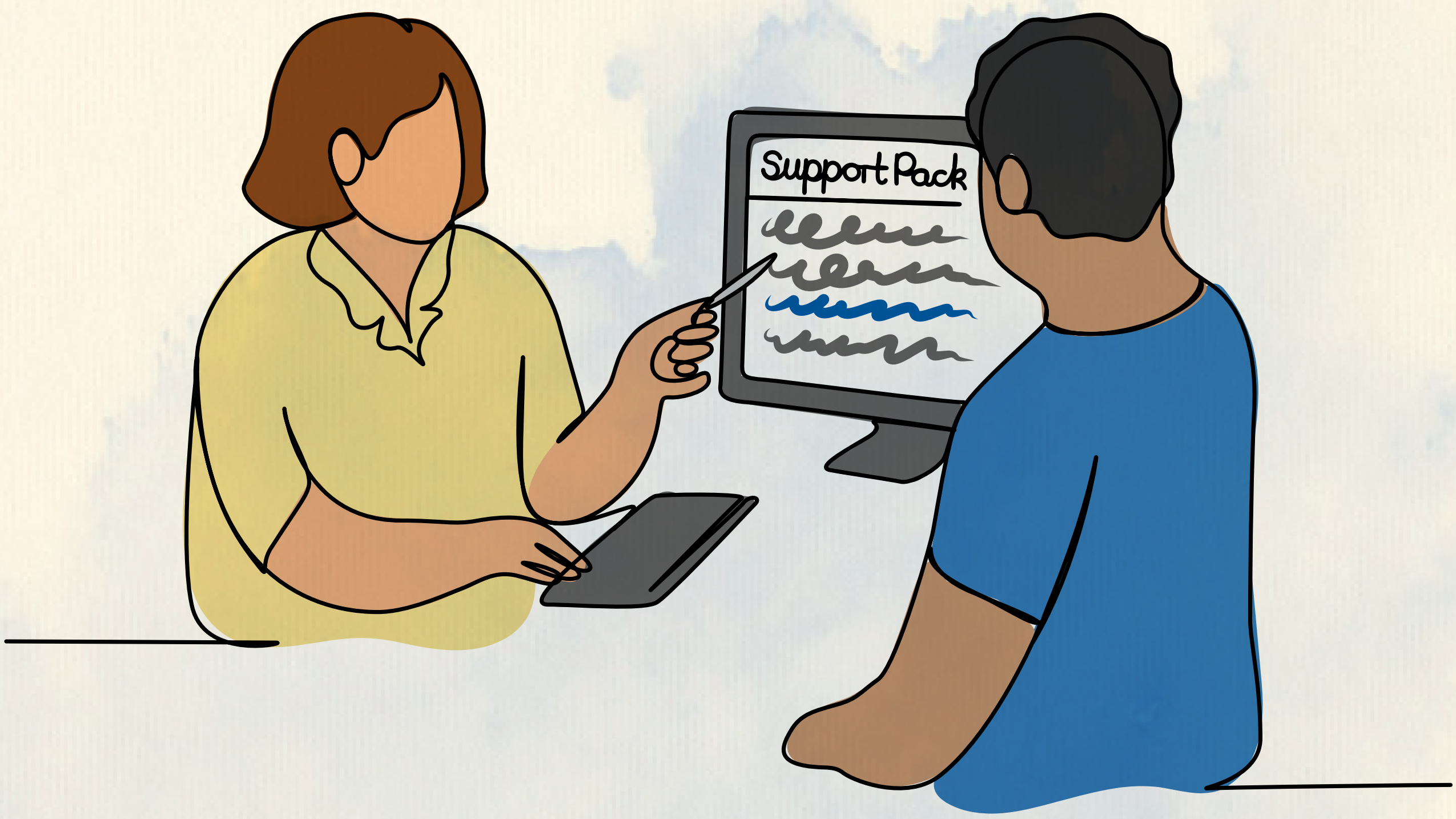

How to make a difference

2. Support phase

The activities outlined in the support phase should only be carried out by those trained in the techniques. However, even if you have not been trained and you are supporting a doctor in this process, it is important that you are aware of what constitutes good practice. It is important to ensure that you are able to signpost to appropriate services, e.g. GP, Occupational Health, counselling, Professional Support Unit. Whilst some aspects of support are important for all counselling/therapeutic interactions, we have developed these principles to address unique features of the professional support context.

2.1 Doctors need to feel a sense of psychological safety in order to engage with professional support. A sense of trust needs to be present in order for doctors to begin to explore the issues they have sought help with.

"I felt very comfortable to be in a private space and just answer questions honestly and go into it. He was very good, he allowed me to talk and develop my thoughts, and asked questions, and I would say it was very helpful." (Resident Doctor)

Recommendations:

- A facilitator who is non-judgmental and supportive is crucial.
- If your role is to support the doctor, reassure them of this so they understand they can trust you and open up.
- Helping the doctor feel valued matters. Affirming their experiences and praising any progress made can help achieve this.
- If professional support is being provided in a performance management context, ensure there is someone acting in a pastoral role with no management or reporting responsibilities to offer the doctor support.
- Take extra care with tone. Ensure that all communication values the doctor's professionalism. For example, small measures such as ensuring their title is correct in initial communications can make a real difference.
- Be clear on the process, on everyone's roles, and what is expected. If comfortable doing so, developing a learning contract can be an effective way of achieving this.
- Peer support is also important, both in terms of helping doctors access information about where to go for support and creating a sense of camaraderie, which may reduce psychological distress.

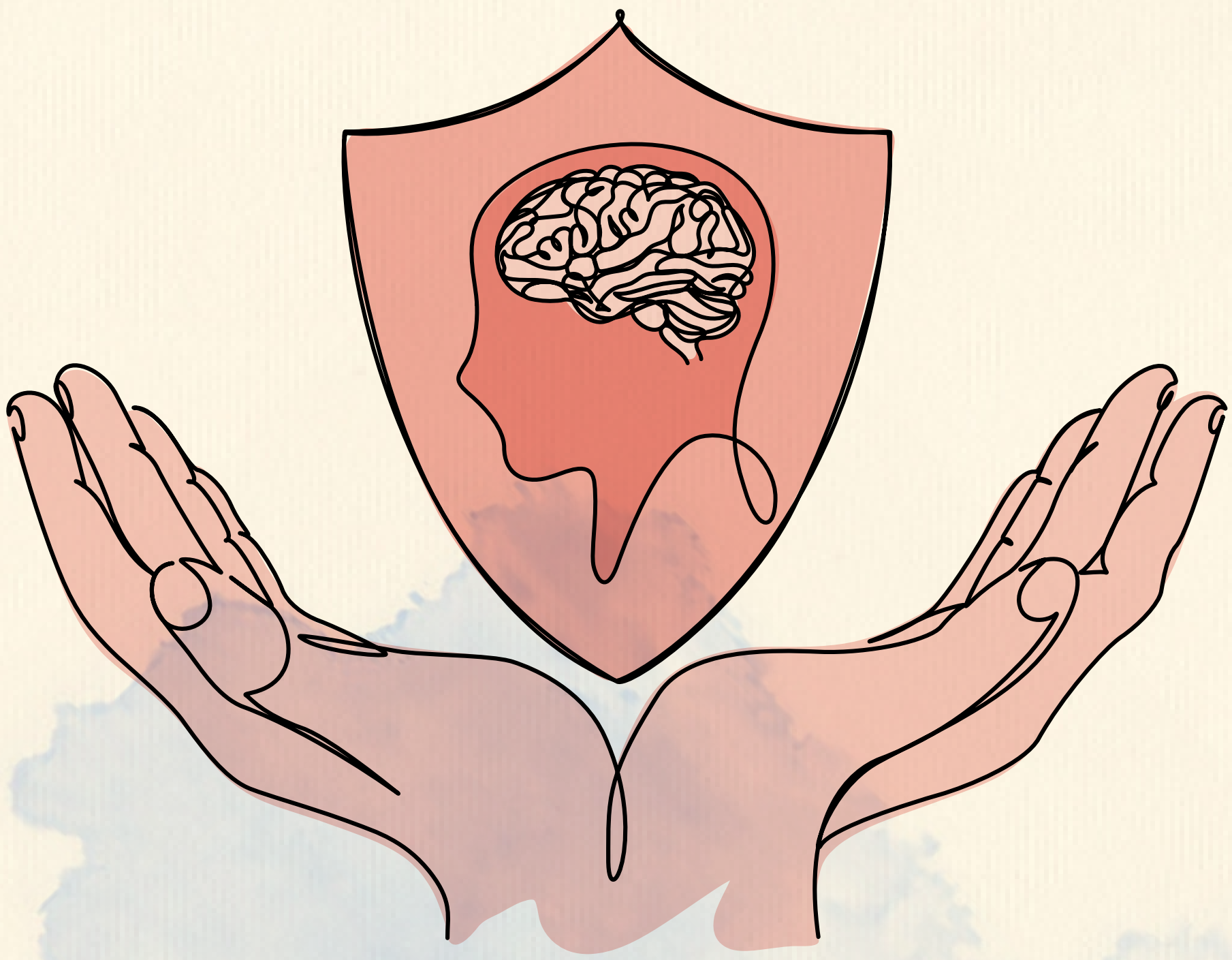

2.2 It is very important for doctors to feel listened to. This allows them to open up and explore any difficulties they may be encountering.

"I think what I really liked about her is that she did pay attention to the things that I said. So having someone that actually paid attention, I felt listened [to], and that was very nice to build up trust with a person." (Resident Doctor)

Recommendations:

- Doctors receiving validation that they have genuine support needs or that they have genuine concerns is an important step in the professional support process.

- Prioritise curiosity and neutrality, and invite thoughts on how others might see the situation.

- Use language that opens up perspectives and possibilities.

- Maintain eye contact and allow doctors to speak without interruption.

- Reflecting back and paraphrasing what people have said shows you're listening, as can using the same language and same metaphors that they do.

- The professional support context may be the first time the doctor has had the opportunity to talk about their experience or the issue they are struggling with. Being able to create a narrative and reflect on why they need professional support may help them to make sense of their experiences.

- Finding ways to minimise a doctor needing to repeat concerns to multiple different people could help them feel listened to and reduce distress.

2.3 Doctors reported that being challenged during the exploration of their situation helped them more fully understand it. This requires a lot of skill and needs to be done in a sensitive way.

"I assume it was just because he was a good counsellor, I felt comfortable talking to him, he was very good at challenging assertions... for example, he would [say]... 'really explain why you think it's like that, explain that to me', so he was very effective in his role." (Resident Doctor)

Recommendations:

- Setting clear expectations can help prepare a doctor to be challenged on their perspective. If the facilitator explains that part of their role is to challenge, the doctor is less likely to be defensive.
- Only challenge when the doctor is ready to receive it. This is a delicate balance, and it may help to ask the doctor if they feel open to being challenged at the beginning or during a session.
- It is important that the facilitator has an in-depth understanding of the professional experience of doctors. This allows the doctor to feel understood.
- If doctors feel they have been wrongly blamed, a trusting, non-judgmental relationship with their facilitator may allow them to explore other perspectives, leading them to engage in the reflective aspects of support.
- It may be helpful to explore the doctor's values with them, to ensure that the goals of professional support are in alignment.

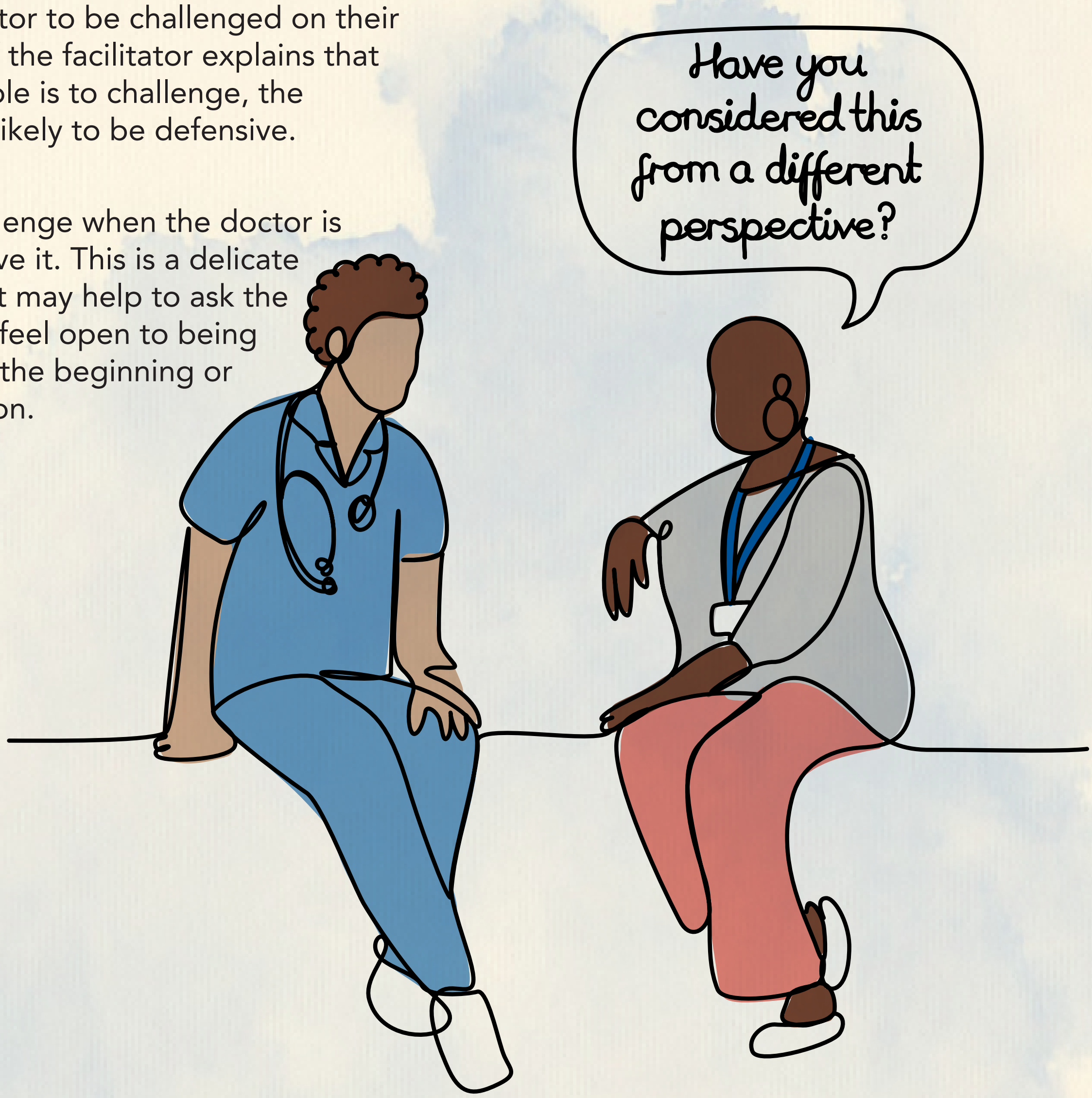

2.4 It is important for doctors to understand the wider context of the difficulties they are experiencing.

"The outlook changes as well, the outlook of 'I'm an anomaly who's not fitting into the training system'... actually, there has been a change in mindset, this is a training system which is imperfect, and I've had to work out the best way of making it work for me." (Resident Doctor)

Recommendations:

- Focus discussion on areas of their professional lives that the doctor can or could control, and help identify those that are outside of their control.

- Encourage doctors to then focus their energy on circumstances that are in their control.

- An example of a tool that can be used to explore this is Circles of Concern and Influence from Stephen R. Covey's The Seven Habits of Highly Effective People. (2020 [1989]) London: Simon and Schuster.

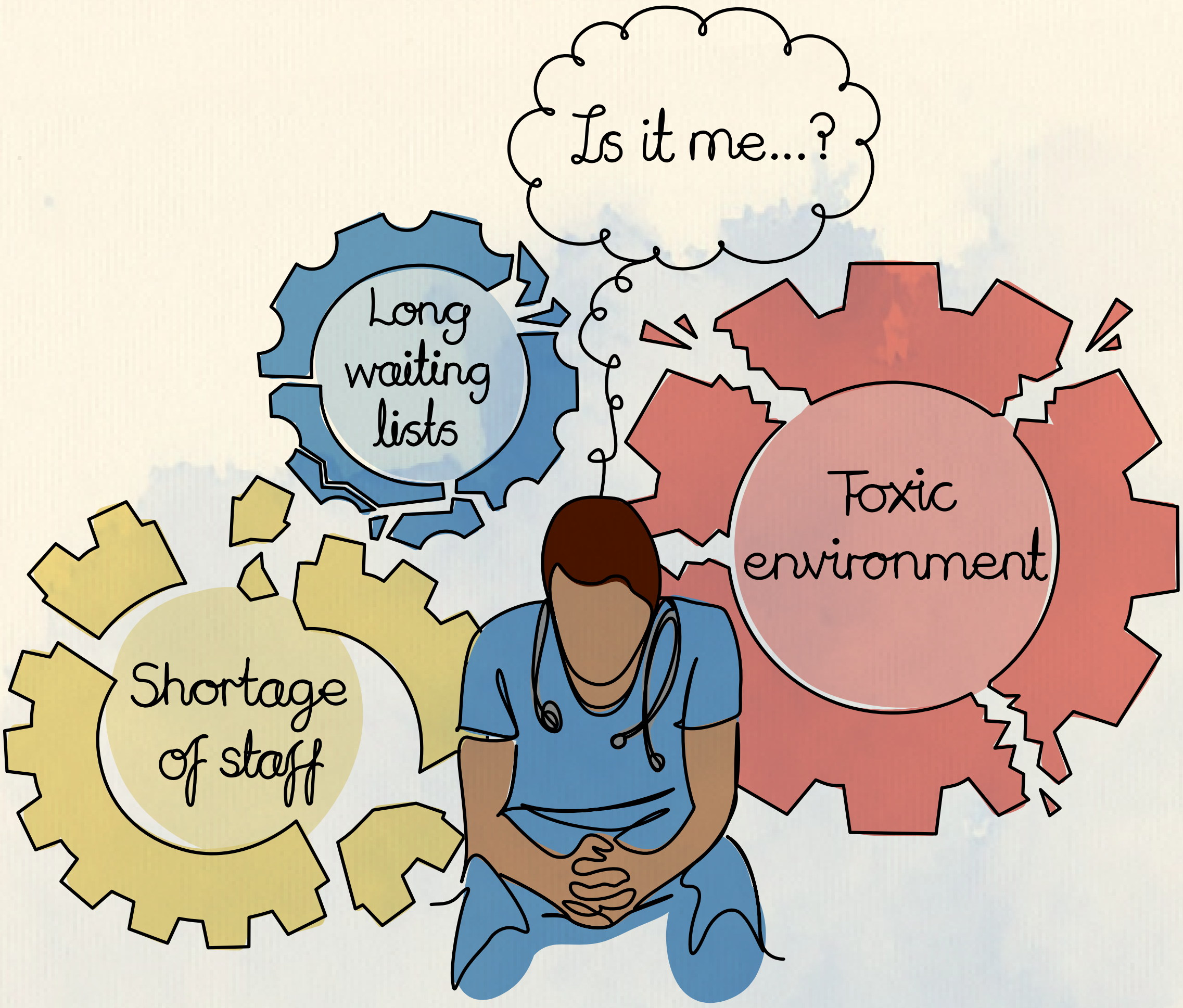

2.5 Sometimes professional support can involve empowering doctors to take control of their experience. This could involve encouraging them to identify their learning needs, to develop new skills, or to identify strengths they already have.

"I think the counsellor at the end [said] 'I don't really need to tell you much cos you know what you've got to do, and that's how we're going to end it.' So... while I probably knew going into it what I wanted to do, the counselling empowered me to do it." (Consultant)

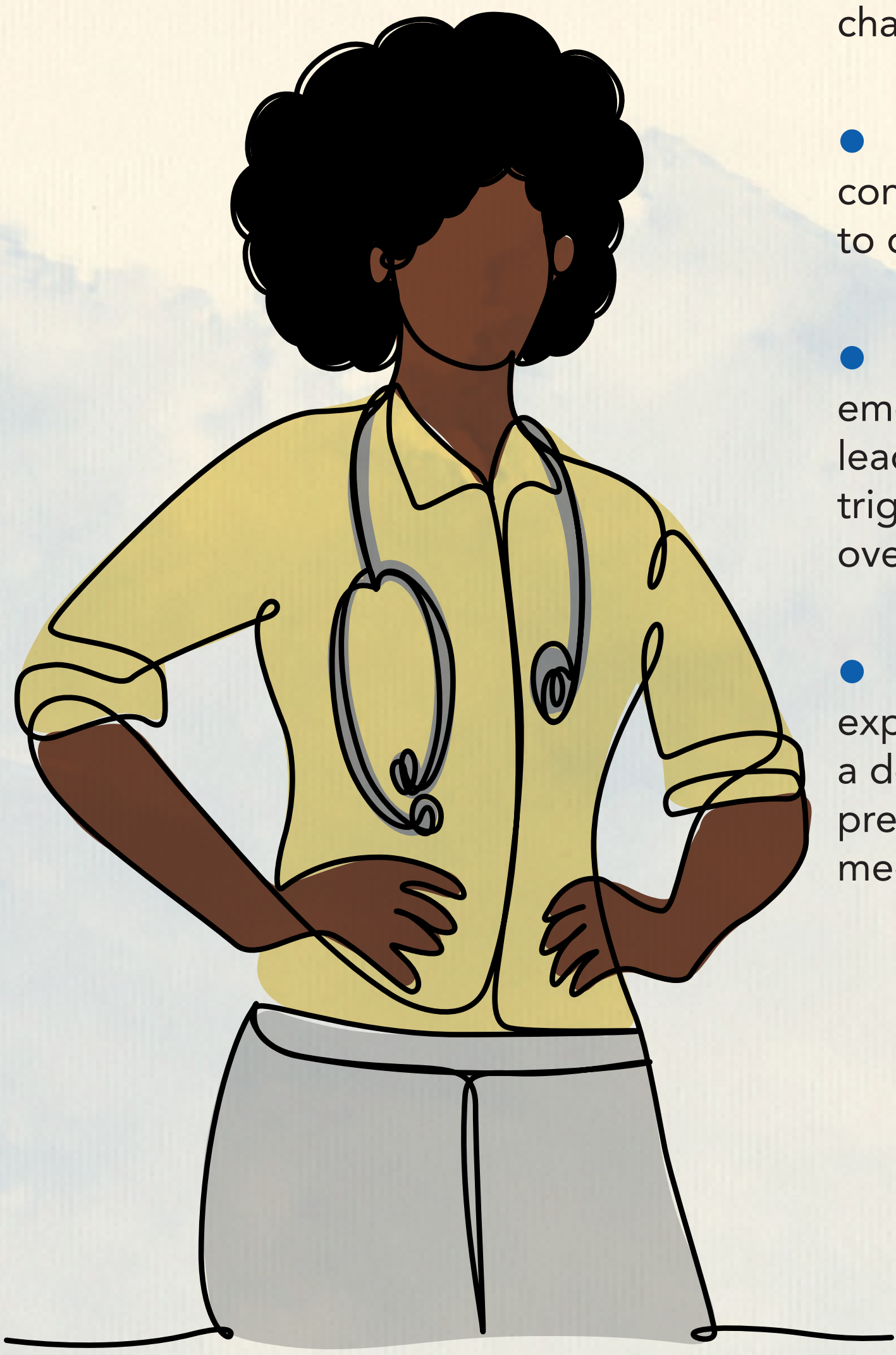

Recommendations:

- Emphasise areas where the doctor is in control of their own development and progression. Even if the need to access support is not the doctor's decision, they can control how they engage and what they get out of the process.
- Encourage a doctor to focus on skills that allow them to exert some control over their situation. An example of this is the skill of negotiation.
- It is important for a doctor to feel a sense of agency when engaging with professional support, and a recognition that change is possible.
- Support doctors to have difficult conversations and communicate their needs to others (through role play, for example).
- Helping a doctor identify their emotional triggers in the workplace can lead to emotional distancing from the triggers so the doctor has greater control over them when they arise.
- If appropriate, it may be helpful to explore alternative career options with a doctor. This can help them feel more prepared if they do decide to leave medicine.

2.6 If a doctor is struggling to understand their role within a situation, a data-driven approach using objective information can help them make sense of this. This helps to make the feedback less personal.

"If I've got external evidence, so if I've got their peer group, particularly if it's their specialist society which has come in and assessed them against a standard, to be able to say: 'look, this isn't my opinion or somebody else's opinion'… So if I've had a review by, say, a Royal College, which then comes back and says, 'actually this is the standard we would expect in that speciality, and you know this is the improvement that needs to be made' really helps with insight." (Professional Support Staff Member)

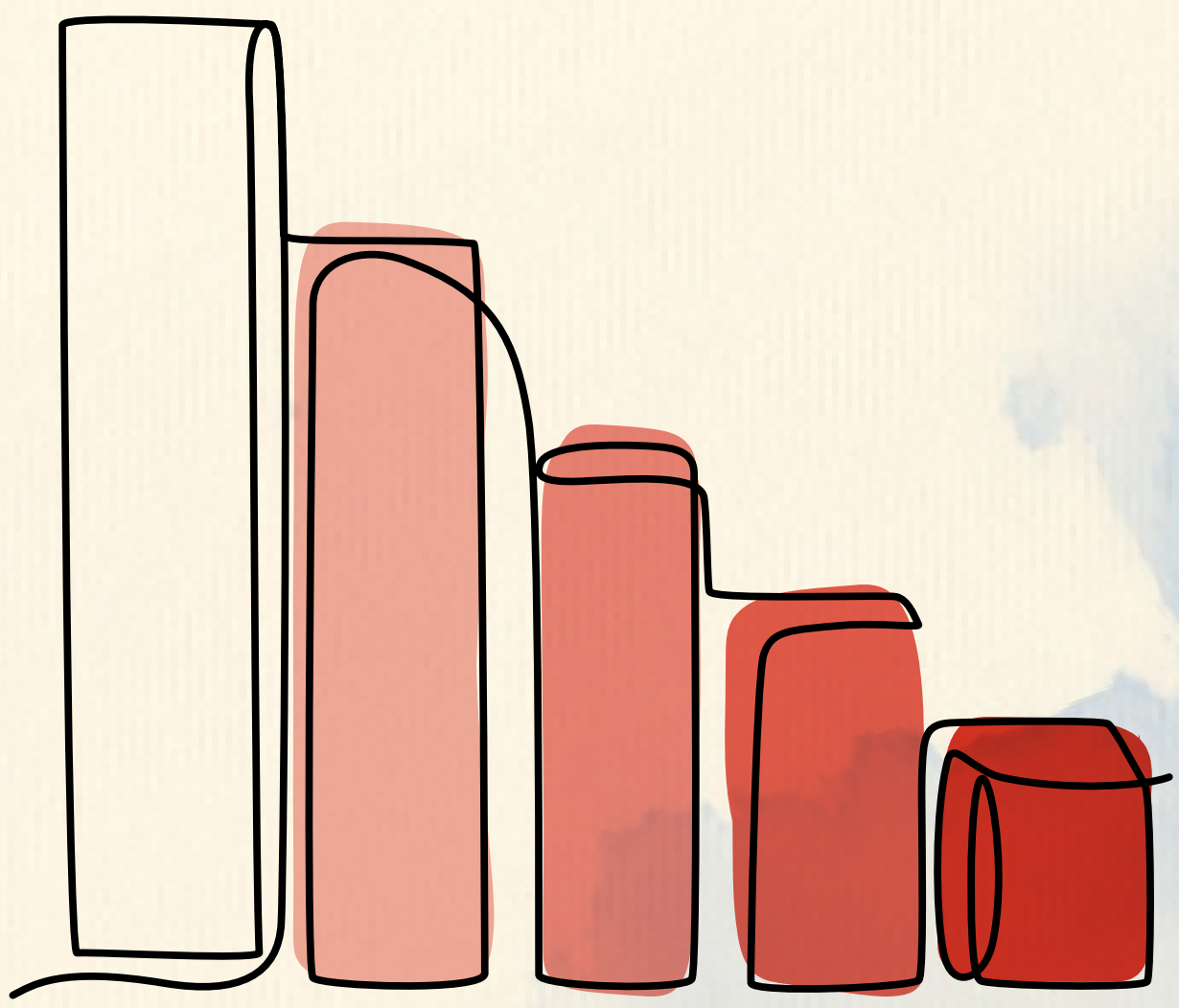

Recommendations:

- Encourage the doctor to choose the feedback they take to the professional support session as this gives them a sense of being in control of their own development and progression. Any major differences between the feedback and the performance concerns raised can be explored.

- Discussing how the situation may have impacted others can invoke empathy in the doctor and make them more open to other perspectives.

2.7 If appropriate, provide well-structured action plans (performance and/or supportive) with a clear, achievable timeline, goals, and roles.

"This was a six-month plan, and month by month they had laid out what needs to happen... so, they asked me to [meet] very specific targets, which are easy to understand because that's exactly like my own clinical practice, how I supervise my trainees. So, the nitty-gritty of the plan was all in there in nice granularity." (Consultant)

Recommendations:

- The actions and responsibilities of the Trust must be clear. Professional support is a two-way responsibility.
- The Professional Support and Remediation provided by NHS Resolution should review any performance action plans developed by Trusts/Integrated Care Systems to ensure that they are well-structured with a clear, achievable timeline, goals and roles.
- Whenever possible, co-create the action plan with the doctor and encourage them to revise it periodically (e.g. timelines).
- Ensuring that the action plan is well-structured with clear goals increases the likelihood that it is achievable. Outcomes also need to be measurable.
- Make sure the roles and responsibilities of all involved in the action plan are clearly articulated.
- Ensure action plans include regular opportunities for constructive feedback.
- Doctors who are completing an action plan need to have a supervisor who can be fair and objective. If relationships within a clinical team have broken down, it is necessary to arrange an external placement for the doctor. If the local Professional Support Unit has been involved in supporting the doctor, they will help to arrange an external placement.
- Ensure action plans are properly implemented, and milestones are assessed by the named individual identified as having responsibility for this role.
- Take into account any illness, neurodivergence or other protected characteristics that may make it difficult for the doctor to make sense of the action plan.
- Ensure that the circumstances of the action plan are kept confidential locally and only shared with those who need to know. It may undermine a doctor if others are aware that they are undertaking professional support.
- If the action plan is compulsory, ensure the doctor is made aware of this.

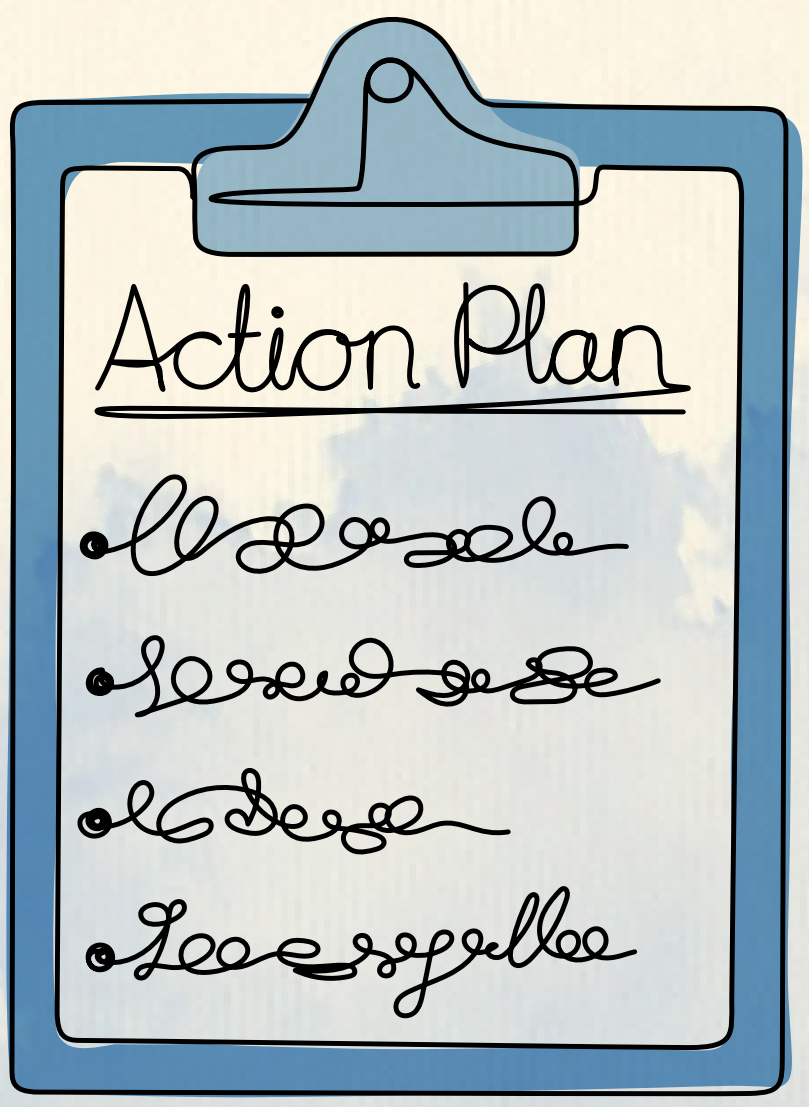

2.8 All doctors accessing professional support need positive relationships to help them engage. Depending on their situation, this can be their relationship with their coach or counsellor, or with a mentor.

"The Trust sent me to a counselling service which was available... they're completely neutral listeners, which was good, it was good to let it all out. So, I had six or more sessions of an hour each, which was very helpful." (Consultant)

Recommendations:

- Ensure any doctor engaging with professional competence interventions also receives parallel wellbeing interventions, such as counselling or mentorship. This should not be provided by their Educational Supervisor or line manager.

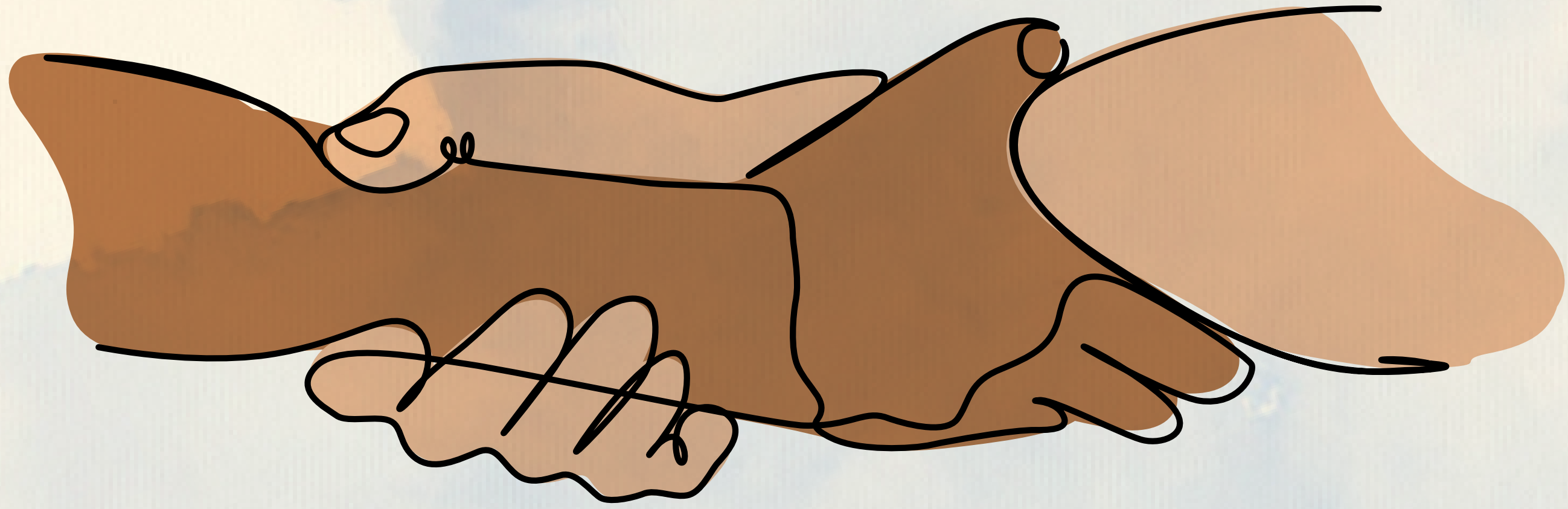

2.9 Informal reflection is crucial in professional support. Encourage doctors to reflect during and following professional support. Keeping a reflective diary can help this process.

"My personal view is that in order to gain the experience from something, you need a period of reflection on it, which may be active and conscious, or may be to some degree subconscious... and that period of reflection allows me a greater understanding, and to gain the experience from whatever it is." (Resident Doctor)

Recommendations:

- Consider introducing doctors to The Academy of Medical Royal Colleges/ COPMeD's Reflective Practice Toolkit (see page 36 for link), which describes the principles for effective reflective practice and includes templates and examples. Another tool that could be used is the GMC's Reflective Practitioner guidance (see page 36 for link).
- It may be helpful to the doctor if they are provided with some questions between professional support sessions to trigger or encourage reflection.
- Encourage doctors to use their e-portfolios or e-appraisal systems, but recognise that some doctors may feel uncomfortable writing honest reflections. It may be helpful for them to use another medium for written reflections.

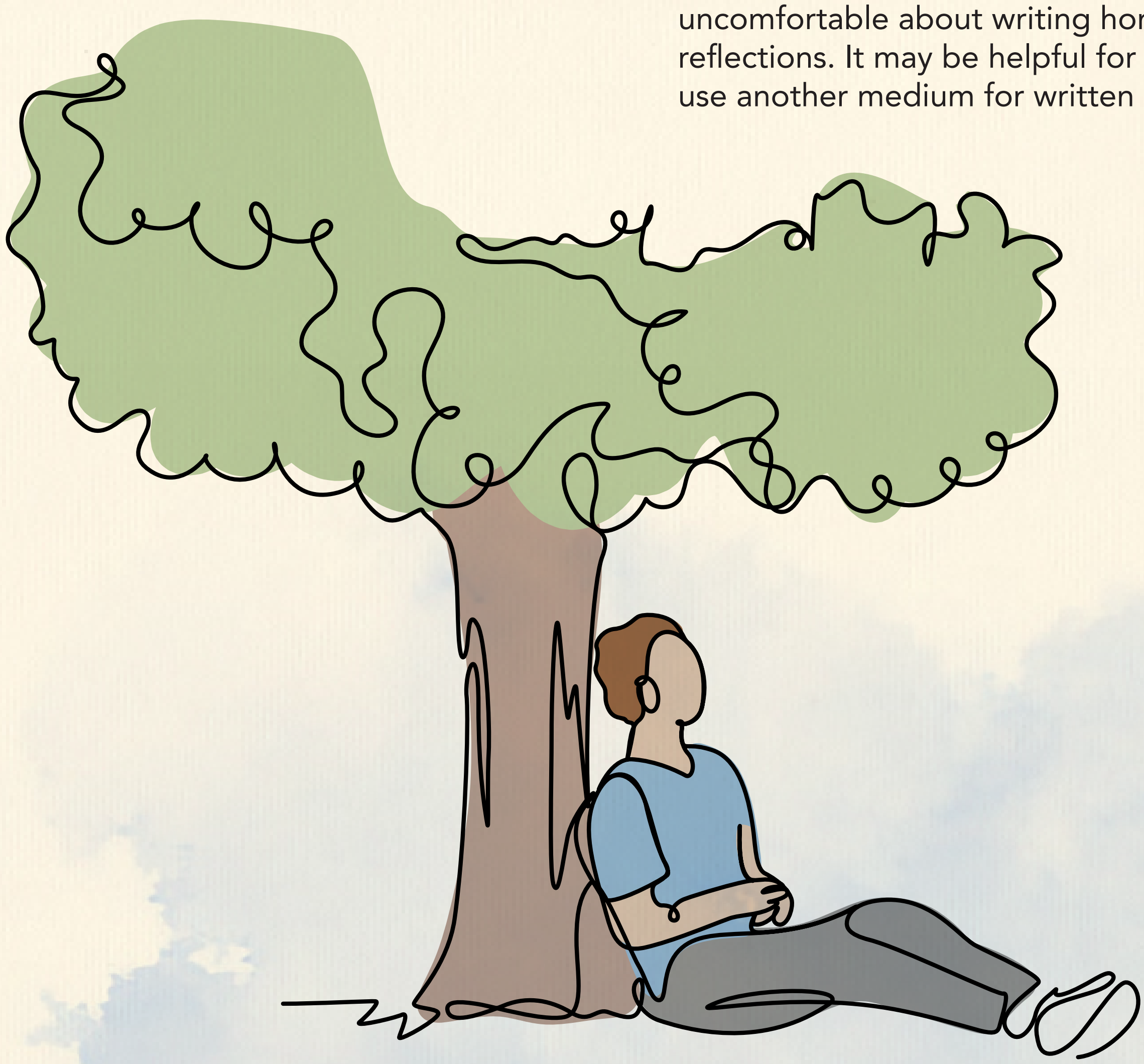

2.10 Doctors may find it helpful to have a written summary of conversations they've had as part of their support.

"One of the things I really struggled with was remembering what we'd talked about and discussed, but what both the coaching person and that initial person did was, after every session would email me a summary of what was discussed, and what they advised that I did in-between, and that was just really helpful and I still go back to."
(Resident Doctor)

Recommendations:

- Provide a short summary of a support session's content, including action points for the doctor.

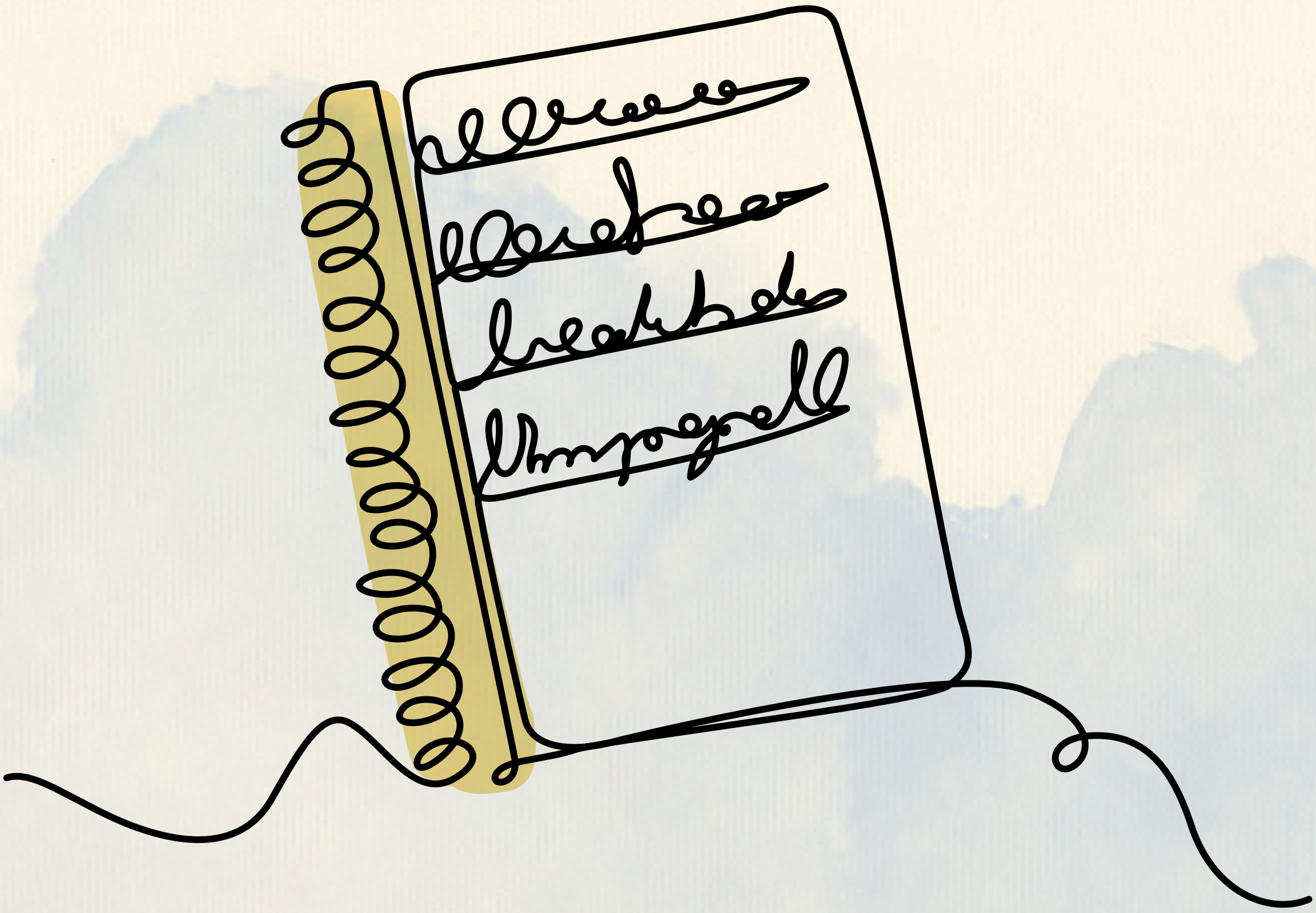

2.11 Different doctors process information in different ways, particularly if they are neurodivergent. It is important to be responsive and adapt to their needs in professional support sessions.

"We spoke about how I absorb information better, and I said I'm a visual person. And I did activities on a piece of paper in front of me and then we'd share them." (Resident Doctor)

Recommendations:

- It is important to tailor support to the doctor's individual circumstances.
- If you are delivering professional support, ask doctors for any feedback on how the session could be delivered more effectively. A doctor may prefer writing or working in a pictorial or more creative style. This could be done at the point of referral.
- If you support doctors in any capacity, ensure that you are trained in recognising and managing neurodivergence, and unconscious bias.
- Ensure doctors feel safe telling you they would prefer to work in a different way.
- If the session is being delivered virtually (e.g. on Zoom or Teams), allow the doctor to have their camera off if they prefer.

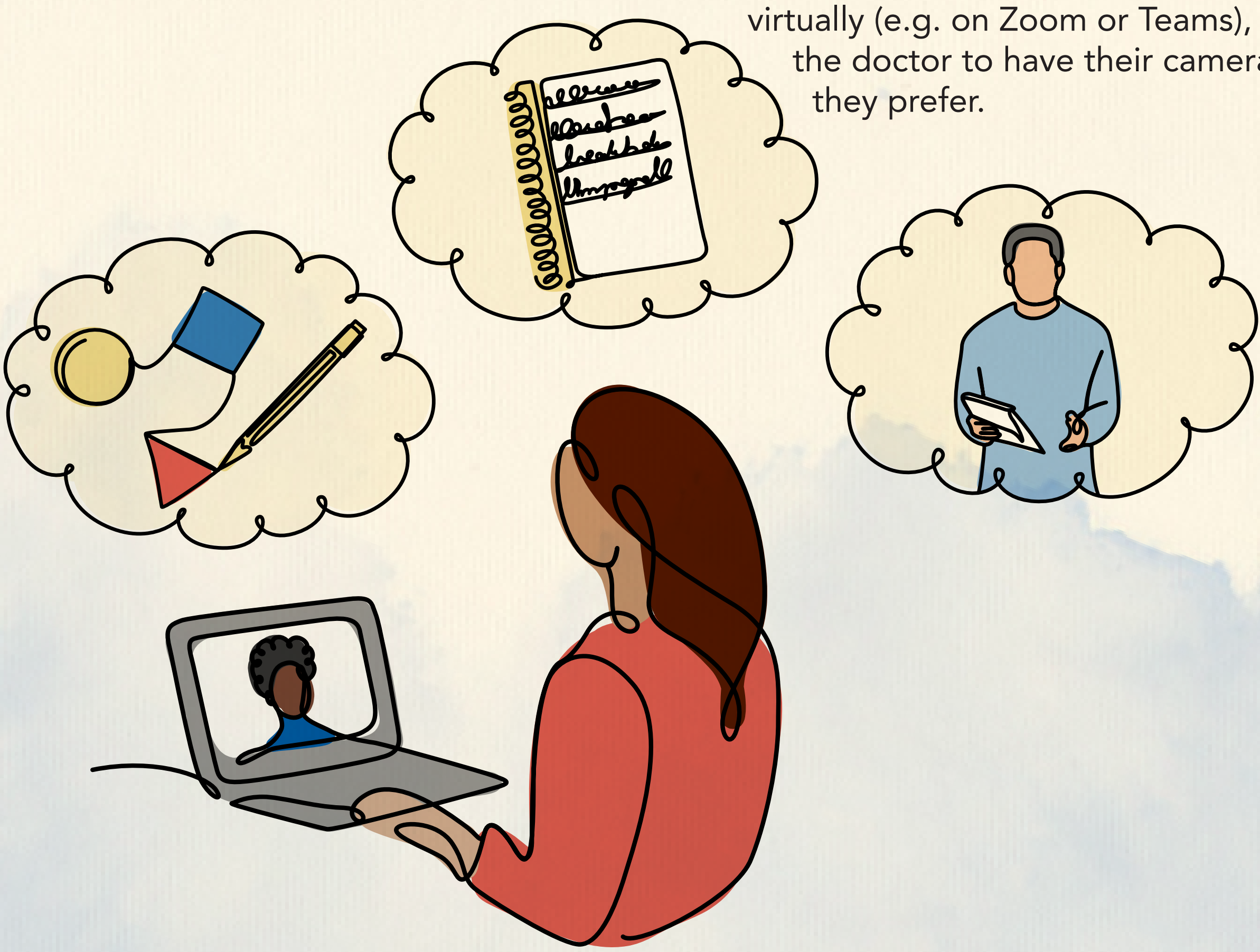

2.12 Doctors have told us that when they have accessed professional support during their working hours, they don't always have a private space or protected time to do this.

"I think the usual stuff is checking that I'm alone, cos obviously everything was online, so like where I was area-wise... a lot of the time they took place during working hours, so it was very difficult to find any empty rooms, so anything like corridors and things, so if people entered, I had to stop talking." (Resident Doctor)

Recommendations:

- Ideally, doctors should be allowed to have a private space and protected time to access support within their workplace.

- Consider offering professional support such as coaching outside of working hours. Doctors may also need some time following the support session to fully reflect on the discussion rather than immediately returning to work.

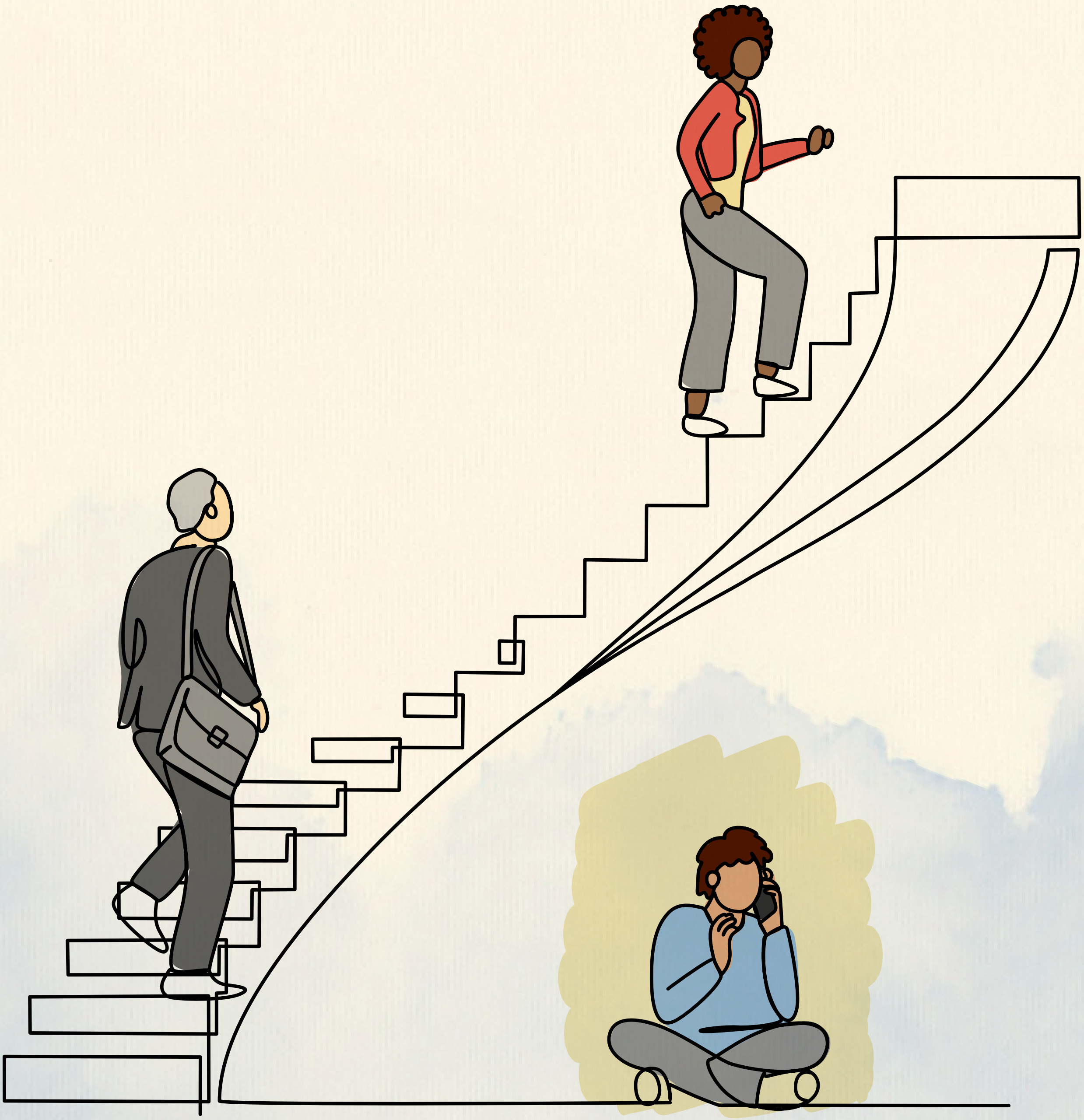

2.13 Doctors have told us that it is important for them to receive the amount of support they require, but it may be restricted by funding.

"It's the limited number of sessions. For example, with the coaching it was just a course of six sessions... So having a bit more freedom in the prescription of what you're allowed to have from the PSU." (Resident Doctor)

Recommendations:

- Try to obtain funding that allows a doctor to access support based on their needs.
- Make doctors aware that professional support is generally a short-term intervention and longer-term support may need to be paid for privately.

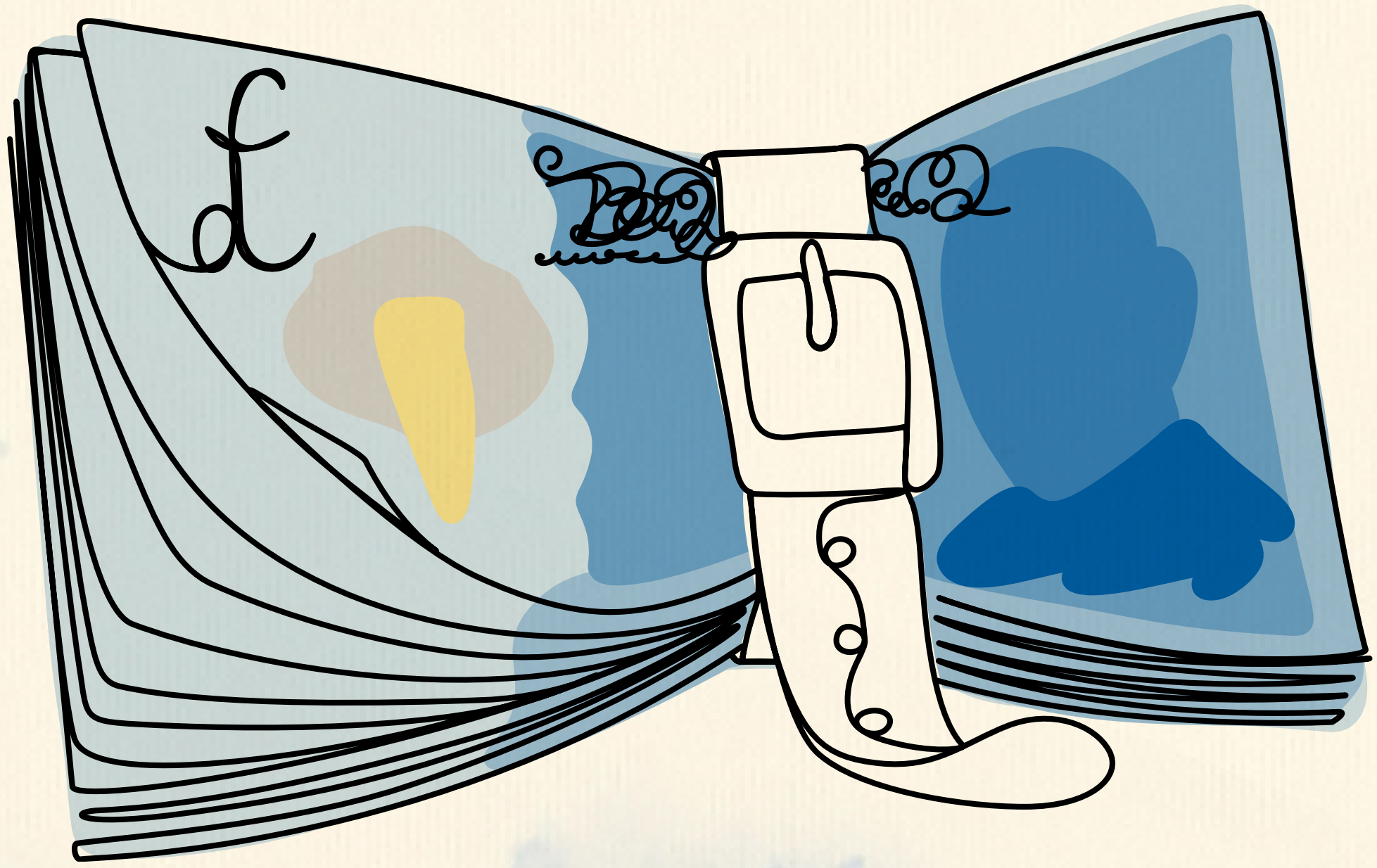

How to make a difference

3. Post-support phase

3.1 In some circumstances, it may be essential for external support services to communicate with a doctor's employing organisation about their support needs.

"If you're completely separated and can't communicate with educators or the Trust, that can be a big disadvantage if people are having serious problems with progression and [educators or the Trust] don't know what we're recommending, and… we're not working together to help the [doctor]. So very often, it's in their interests that we should share information, if they'll agree to it." (Professional Support Staff Member)

Recommendations:

- Check with the doctor that permission has been granted for specific information about them to be shared.
- Communication between two organisations needs to be clear, with the correct personnel identified.
- Only share what is necessary.
- Remember that the importance of psychological safety continues even after professional support has ended. None of the communications should undermine this.

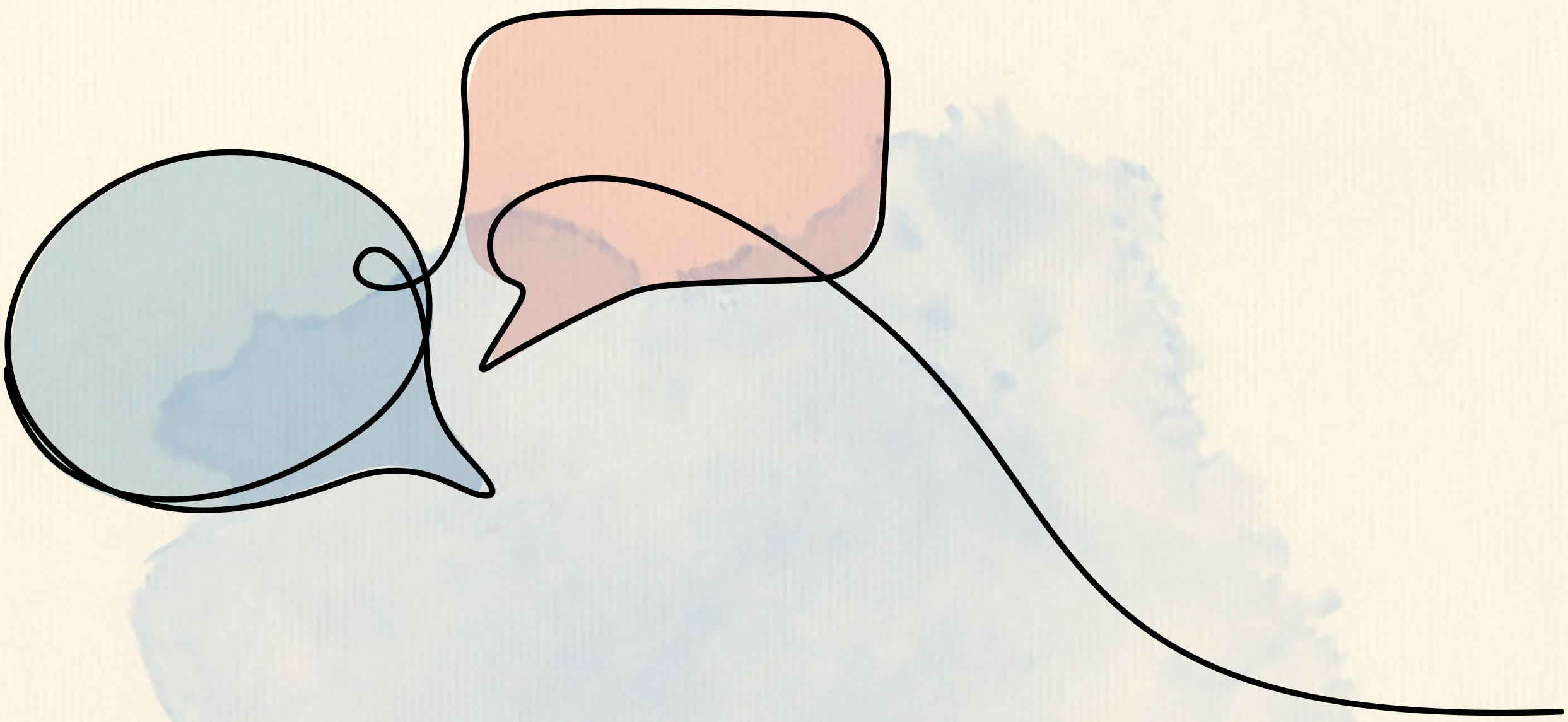

3.2 It is important for doctors to be able to appropriately access follow-up support in future if it's needed once their professional support has concluded.

"I think that was really useful… being a bit proactive about looking at what's happening in different parts of my life, and what I'm in control of and what I'm not, I think that history helped me to… seek help quickly." (Resident Doctor)

Recommendations:

- Support doctors to recognise the signs that they are struggling and need further support.
- It may be helpful to signpost support to doctors and provide them with contact information for support services.
- Be aware of periods when doctors may need extra support. Key transition points, such as moving between core and specialist training, and becoming a consultant, are examples of periods when doctors may experience difficulties.

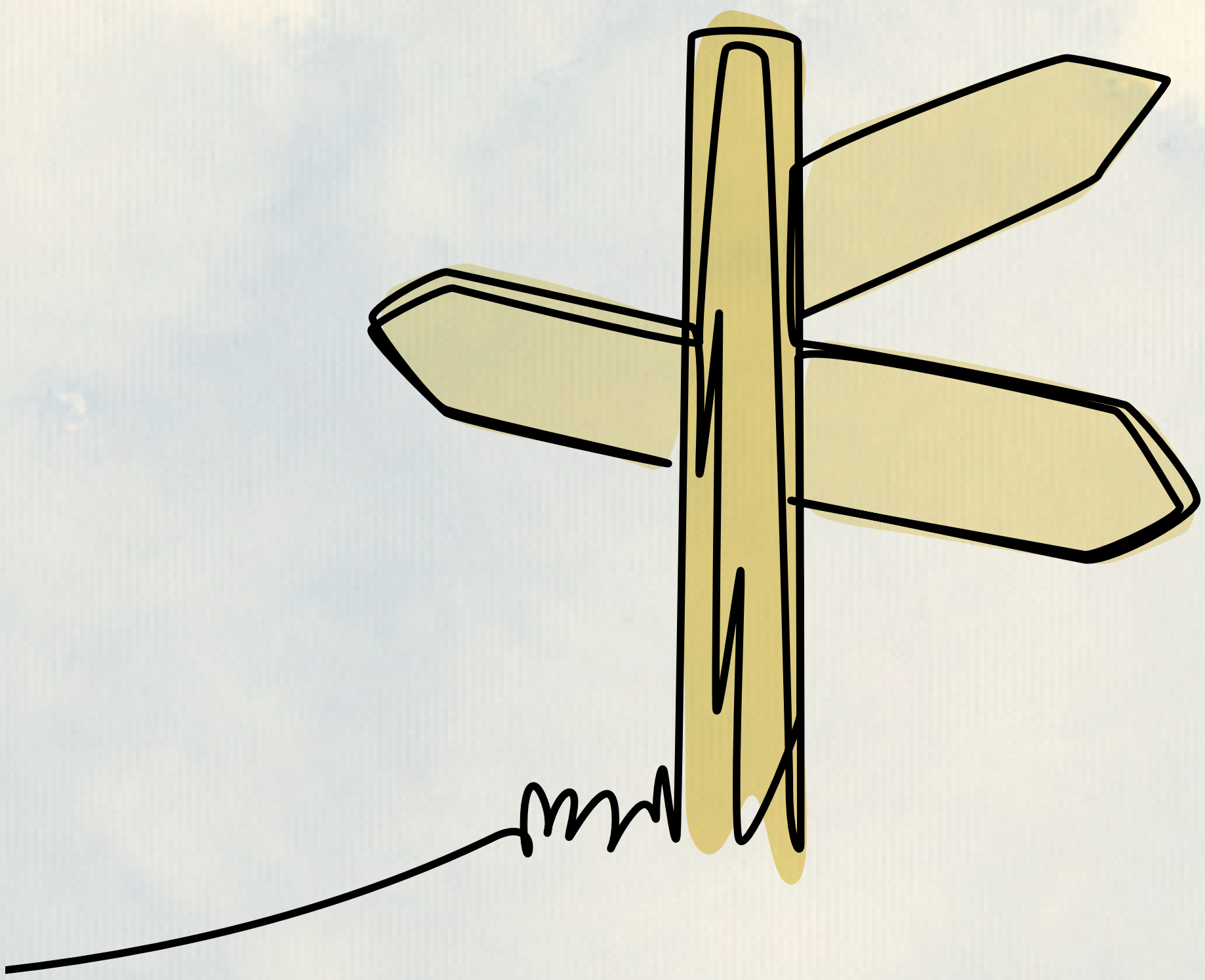

3.3 Professional support staff should be open to feedback from doctors in order to optimise the delivery of the service.

"When you're promoting engagement, it's important that you get some idea of what their viewpoint is, and it helps that even if we can't always accommodate what they want, at least we're able to listen to what their issues are and take that into account." (Professional Support Staff Member)

Recommendations:

- Ensure there are processes in place that allow doctors to feedback in ways that feel safe to them.

- If your service has the resources and freedom to make changes, try to be responsive to what doctor's feed back to you. Communicate with doctors about the changes that have been made.

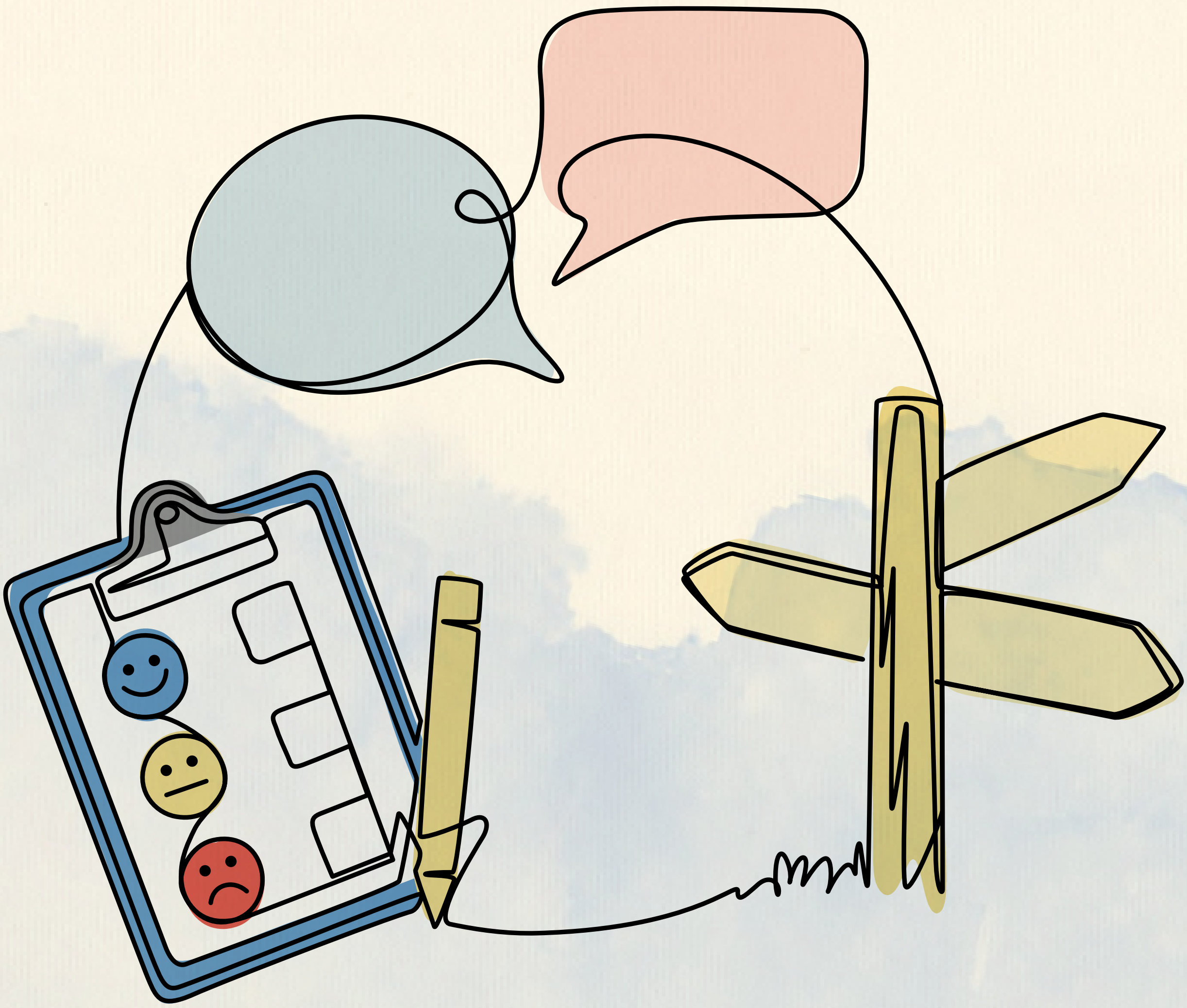

Find out more

To read our other publications, visit our website at:

www.plymouth.ac.uk/research/restore

Contact the authors at:

nicola.brennan@plymouth.ac.uk

ellie.reynolds@plymouth.ac.uk

Acknowledgements

We would like to sincerely thank all those who supported this study – specifically, our study participants and our stakeholder group, which involved professional and patient representatives, who so generously gave their time and expertise, even when this was personally psychologically difficult.

Funder statement

This project was supported by the NIHR HSDR programme with grant number NIHR130922. The views and opinions expressed herein are those of the authors and do not necessarily reflect those of the NIHR or HSDR programme.

How to reference this document

Brennan N, Reynolds E, Price T, Wong G, Withers L, Cleland J, Lloyd H, Gale T (2025) A Guide to Optimise the Delivery of Professional Support in the NHS. University of Plymouth, Plymouth. **www.plymouth.ac.uk/research/restore**

Find out more

The following sources of support can be used to signpost or refer doctors

Trust-based:

- Employee Assistance Programme
- Postgraduate Medical Education Centre
- SAS Advocate role www.nhsemployers.org/system/files/2022-09/SAS-Advocate-role-guidance.pdf
- Occupational Health Physician
- Peer support groups – there may be peer support groups set up within the Trust or area.

External:

- Professional Support Unit or Professional Support and Wellbeing Services
- Practitioner Health www.practitionerhealth.nhs.uk
- Doctors in Distress doctors-in-distress.org.uk
- BMA www.bma.org.uk/advice-and-support/your-wellbeing
- Hospital Consultants and Specialists Association www.hcsa.com
- Your NHS GP
- Practitioner Performance Advice resolution.nhs.uk/services/practitioner-performance-advice

Find out more

Links

Page 14: NHS tool to improve readability
readability.ncldata.dev

Page 26: The Academy of Medical Royal Colleges/COPMeD's Reflective Practice Toolkit
www.aomrc.org.uk/wp-content/uploads/2018/09/Reflective_Practice_Toolkit_AoMRC_CoPMED_0818.pdf

GMC's Reflective Practitioner Guidance
www.gmc-uk.org/education/standards-guidance-and-curricula/guidance/reflective-practice/the-reflective-practitioner---guidance-for-doctors-and-medical-students

UNIVERSITY OF PLYMOUTH

RESTORE

CAMERa

UNIVERSITY OF OXFORD

NANYANG TECHNOLOGICAL UNIVERSITY SINGAPORE

Document design and illustration by www.ccanimationstudio.co.uk
